# Elevated Tumor-Associated Androgen Receptor Activity Correlates with Poor Immune Infiltration and Immunotherapy Response across Cancer Types

**DOI:** 10.1158/2767-9764.CRC-25-0409

**Published:** 2026-01-05

**Authors:** Ya-Mei Hu, Faming Zhao, Julie N. Graff, Canping Chen, Yi Zhang, Jayne M. Stommel, Jinho Lee, Gabriel M. Zangirolani, Joshua Rose, George V. Thomas, Hui Wu, Adel Kardosh, Gordon B. Mills, Joshi J. Alumkal, Amy E. Moran, Zheng Xia

**Affiliations:** 1 Department of Biomedical Engineering, Oregon Health & Science University, Portland, Oregon.; 2 Knight Cancer Institute, Oregon Health & Science University, Portland, Oregon.; 3 VA Portland Health Care System, Portland, Oregon.; 4 Knight Diagnostic Laboratories, Oregon Health & Science University, Portland, Oregon.; 5 Department of Pathology and Laboratory Medicine, Oregon Health & Science University, Portland, Oregon.; 6 Division of Biomaterial and Biomedical Sciences, Department of Oral Rehabilitation and Biosciences, Oregon Health & Science University, Portland, Oregon.; 7 Division of Oncological Sciences, Oregon Health & Science University, Portland, Oregon.; 8 Department of Cell, Developmental and Cancer Biology, Oregon Health & Science University, Portland, Oregon.; 9 Department of Internal Medicine, Rogel Cancer Center, University of Michigan, Ann Arbor, Michigan.; 10 Center for Biomedical Data Science, Oregon Health & Science University, Portland, Oregon.

## Abstract

The role of androgen receptor (AR) signaling in modulating antitumor immune responses has received increasing attention in recent years; however, its broader impact across diverse cancer types and between sexes remains largely unexplored. In this study, we investigated how AR activity correlates with tumor-infiltrating leukocytes, patient prognosis, and immunotherapy response across cancers and sexes. We inferred AR activity using a network-based approach across bulk RNA sequencing [RNA-seq; The Cancer Genome Atlas (TCGA)], single-cell RNA-seq (prostate cancer meta-atlas), and immunotherapy cohorts. Pathway analysis and Cox regression assessed mechanisms and survival. Immune infiltration and signatures were evaluated via TIMER and single-sample gene set enrichment analysis. Key findings were validated using digital spatial profiling and IHC. Our pan-cancer analysis of 33 TCGA cancer types revealed broad variability in AR activity, with highest observed in prostate adenocarcinoma. Genes significantly correlated with AR activity showed negative associations and were enriched in immune activation pathways. Notably, AR activity inversely correlated with leukocyte abundance and IFNγ pathway activity across tumors and sexes—unlike estrogen or progesterone receptors. Longitudinal biopsy analysis in metastatic prostate cancer showed that AR inhibition enhanced immune cell and IFNγ signatures. Single-cell analysis confirmed that tumor-intrinsic AR activity inversely correlates with immune infiltration in prostate cancer. Furthermore, low AR activity is significantly associated with favorable immunotherapy responses in hormone-independent cohorts. Spatial proteomics showed a negative correlation between AR and CD45 protein in sarcoma and ovarian cancers. These findings suggest AR activity as a pan-cancer predictive biomarker of immunotherapy response and support that AR blockade in immunotherapy-refractory tumors represents a promising treatment strategy, regardless of tumor type or patient sex.

**Significance:**

Tumor-associated AR activity negatively correlates with immune infiltration and immunotherapy response across cancers, independent of sex, suggesting that combining AR inhibitors with checkpoint blockade may benefit patients with immunotherapy-refractory tumors.

## Introduction

The androgen receptor (AR) is a nuclear transcription factor (TF) that mediates the biological effects of androgens, such as dihydrotestosterone and testosterone (1). Upon ligand binding, AR translocates to the nucleus and regulates the transcription of target genes through androgen response elements (2). Androgen/AR signaling plays an essential role in reproductive development in both sexes (3, 4). Beyond its roles in development and endocrine function, AR is expressed in a wide range of cells and tissues—including the prostate, muscle, adipose tissue, bone, and cells of the cardiovascular, neural, and immune systems—and exerts significant physiologic effects (4, 5). Androgen/AR signaling is widely recognized as a critical driver of hormone-related malignancies, particularly prostate cancer in which AR activation is a key oncogenic driver, and androgen deprivation therapy (ADT) remains a cornerstone of treatment (6).

In the past 3 years, there has been a growing interest in the immunomodulatory role of androgens within the immune system in both health and disease (7, 8). In the context of cancer and the tumor microenvironment (TME), AR is expressed in various immune cell types, including neutrophils (9), macrophages (9), T cells (10, 11), and B cells (12), and contributes to the regulation of both innate and adaptive immune responses. Recent studies have highlighted a critical role for AR signaling in suppressing antitumor immunity in and beyond reproductive cancers. For example, we reported that AR represses *Ifng* expression in T cells, and AR inhibition with enzalutamide (enza) restored IFNγ production, thereby enhancing the efficacy of anti–PD-1 therapy in metastatic castration-resistant prostate cancer (mCRPC; ref. 11). In preclinical models of bladder, colon, melanoma, and liver cancer, T cell–intrinsic AR signaling has been shown to promote CD8^+^ T-cell exhaustion in males, contributing to sex-based disparities in antitumor immunity (13, 14). More recently, we also demonstrated that AR directly represses MHC class I expression in prostate cancer, uncovering a tumor-intrinsic mechanism of an oncogene driving tumorigenesis while promoting immune evasion (15). Together, this work suggested that perhaps AR signaling might also limit immune infiltration in AR-responsive tumors, underscoring the need for comprehensive analyses to better define its immunologic impact.

In this study, we conducted a pan-cancer analysis of sex hormone nuclear receptors, including the AR, estrogen receptors (ER), and progesterone receptor (PR), with extended analysis of AR given our previous findings (11, 15). Leveraging a network computational approach, we comprehensively investigated the relationship between AR activity and tumor-infiltrating leukocytes across various cancer types and within sexes. Using bulk RNA sequencing (RNA-seq) data from The Cancer Genome Atlas (TCGA), we evaluated sex hormone receptor activity across 33 cancer subtypes, assessed associations with patient survival, and examined correlations with tumor-infiltrating leukocyte abundance and immune-related gene signatures. Utilizing a curated mCRPC cohort, we compared AR activity and leukocyte profiles at baseline and following AR inhibitor treatment. We also identified an association between AR activity and tumor immunotherapy response using six independent, publicly available clinical datasets. Finally, using a curated digital spatial profiling (DSP) protein dataset and IHC from real-world patient biopsies available through our Serial Measurements of Molecular and Architectural Responses to Therapy (SMMART) clinical program, we demonstrate that AR protein levels inversely correlate with leukocyte abundance in the TME. Together, these findings reveal a robust, sex-independent negative association between AR activity and tumor immune infiltration and immunotherapy response. This work suggests that combining AR signaling inhibitors with checkpoint blockade may be especially effective in tumors with elevated AR activity.

## Materials and Methods

### Data source and processing

Bulk RNA-seq data and corresponding clinical data for 33 cancer types from TCGA consortium were retrieved from The NCI Genomic Data Commons (GDC; https://portal.gdc.cancer.gov/) via the TCGAbiolinks R/Bioconductor package (version 2.24.1; RRID: SCR_017683; refs. 16–18). The Genotype-Tissue Expression (GTEx) data were downloaded from the portal (https://www.gtexportal.org/home/downloads/adult-gtex#bulk_tissue_expression; RRID: SCR_013042; ref. 8). The independent datasets of clinical trials were accessed from the Gene Expression Omnibus (GEO; https://www.ncbi.nlm.nih.gov/geo/; RRID: SCR_005012) database [GSE145996 (19), GSE135222 (20), and GSE126044 (21)], the database of Genotypes and Phenotypes (dbGaP; https://www.ncbi.nlm.nih.gov/gap/; phs000452.v2.p1; ref. 22), or publicly available source data [Cindy Yang and colleagues (23), Westbrook and colleagues (24), and Guan and colleagues (11)]. The R package “edgeR” (version 3.38.1; RRID: SCR_012802) was utilized to process the downloaded bulk RNA-seq data. The downloaded RNA-seq data were then converted into transcripts per kilobase million (TPM) values and further transformed as log (1 + TPM) for downstream analyses (25–27). Three independent single-cell RNA-seq datasets were collected from previous publication [Zhang and colleagues (28), Zheng and colleagues (29), and Hawley and colleagues (30)].

### Estimating single-sample TF activity levels

To measure AR, ER, and PR activity of each sample from datasets, single-sample Virtual Inference of Protein activity by Enriched Regulon analysis (VIPER) was performed using the VIPER R package (version 1.30.0; ref. 31). A log1p-transformed TPM gene expression matrix and a regulatory network were used as inputs for VIPER analysis. The TF regulons (the regulatory network) used in this study were curated from several databases as previously described (32).

### Survival analysis

Survival analysis was performed using progression-free interval (PFI) endpoints across TCGA cohorts for hormone receptors. For AR activity, both PFI and overall survival (OS) outcomes were evaluated. The selection of endpoints was based on recommendations from a published article (33), which indicated that OS endpoints were not recommended for multiple cancer types because of a small number of events and the need for a longer follow-up period. Univariate Cox proportional hazards models were used to assess the association between AR activity levels and clinical outcomes in each cancer type. *P* values less than 0.05 were considered statistically significant. Of note, acute myeloid leukemia (LAML) lacks PFI data, and the PFI endpoint in pheochromocytoma and paraganglioma (PCPG) was not recommended in a previous study for the same reasons (33).

### Identification of AR activity–correlated genes and overrepresentation analysis

To identify genes commonly correlated with AR activity in 33 TCGA cancer types, we performed Pearson correlation analysis to assess the correlations between AR activity and each gene expression in each cancer type. We then overlapped the significantly correlated gene lists in the 33 cancer types to identify the 31 genes common in all 33 TCGA cancer types.

The “clusterProfiler” R package (version 4.4.1; RRID: SCR_016884) was used to perform Gene Ontology (GO) biological process (BP) and Reactome overrepresentation analysis for the exploration of pathways and biological processes relating to the 31 AR activity–correlated genes (34, 35). Enrichment pathways and categories at adjusted *P* value < 0.05 are selected as statistically significant and the top 15 significantly enriched GO BP or Reactome categories are visualized in the dot plots.

### Estimation of tumor-infiltrating immune cells

Tumor IMmune Estimation Resource 2.0 (TIMER 2.0; http://timer.cistrome.org/; RRID: SCR_018737) web server is a comprehensive resource for evaluating the abundances of tumor-infiltrating immune cells across diverse cancer types (36–38). We used it to obtain the infiltration levels of B cells, CD4^+^ T cells, CD8^+^ T cells, neutrophils, macrophages, and myeloid dendritic cells in 32 cancer types from TCGA database estimated by the TIMER immune deconvolution algorithm. Alternatively, we acquired gene signatures of 28 immune-related cell types for further analysis of immune cell composition in tumors (39). The single-sample gene set enrichment analysis (ssGSEA; RRID: SCR_026610) implemented in the GSVA R package (version 1.44.1; RRID: SCR_021058) was performed to calculate sample-wise absolute enrichment scores of these signatures in each sample (40). The ssGSEA algorithm is a rank-based method to assess the expression levels of genes within a gene signature against all other genes in each sample within a given dataset. Log-transformed gene expression profiles (GEP) and gene sets from published studies were used as input to ssGSEA.

### Gene signature score calculation

We collected three published IFNγ-related gene expression signatures and one tertiary lymphoid structure (TLS) signature associated with prognostic value to immunotherapy, including Hallmark IFNγ response gene set (IFNγ MSigDB Hallmark gene set; ref. 41), 18-gene T cell–inflamed GEP (42), the immunologic constant of rejection (ICR) 20-gene signature (43), and the TLS signature (Cabrita and colleagues; ref. 44). Gene lists of each signature are in Supplementary Data S1. The activity of these gene signature scores was assessed using ssGSEA (RRID: SCR_026610) implemented in the R package GSVA (version 1.44.1; RRID: SCR_021058; ref. 40).

### Correlation analysis

We computed the correlation between two continuous variables using Pearson correlation coefficients. A significance threshold of *P* < 0.05 (Pearson correlation test) was applied to determine the significance of correlation. When calculating the correlation between immune pathways and AR activities, the overlapping genes between the gene signatures and the AR regulon were removed from the pathway gene sets.

### Master regulator analysis and pathway analysis

RNA-seq data of 21 matched tumor biopsy samples of prior to enza and at the time of progression (baseline and progression tumors; ref. 24) were used to evaluate differential TF activities and to perform IFNγ signatures, immune cell signatures, and GO BP gene set enrichment analysis (GSEA; RRID: SCR_003199; ref. 45). Differential gene expression analysis was first performed using DESeq2 (version 1.32.0; RRID: SCR_015687; ref. 46). Gene expression differences were considered significant when adjusted *P* values were < 0.05. TF activity was inferred by msVIPER algorithms provided in the VIPER R package (version 1.26.0; ref. 31). The Wald test statistic results from DESeq2 output served as a gene list input data for VIPER analysis. The transcriptional regulatory network used in this study was curated from four databases as previously described (32). Pathway analysis was performed using the GSEA function implemented in the “clusterProfiler” R package (version 4.4.1; RRID: SCR_016884; refs. 34, 35). The Wald test statistic results from DESeq2 output served as a preranked gene list input of GSEA. The gene sets were considered to be activated if their adjusted *P* value was less than 0.05 and the normalized enrichment score was greater than 0.

### Single-cell analysis methodology

The recently published human prostate cancer single-cell RNA-seq (scRNA-seq) meta-atlas (28) and the scRNA-seq dataset (30) were reanalyzed using the Seurat pipeline (v4.1.0; RRID: SCR_007322). Briefly, the BBKNN algorithm was applied to mitigate potential batch effects. Uniform Manifold Approximation and Projection with the Leiden algorithm was employed for clustering and visualizing single-cell distribution (47). Cell clusters were annotated based on previously reported cell type marker genes of human prostate cancer (28) and the combined automatic annotation method CellTypist (RRID: SCR_024893; ref. 48).

### Data analysis of the independent immune checkpoint blockade datasets

To further investigate the clinical utility of single-sample AR activity in different cancer types, we obtained five independent datasets from published studies and one of our studies with bulk RNA-seq data for tumor samples collected from patients with clinical information about immune checkpoint inhibitor (ICI) therapeutic responses. We used baseline gene expression profiles for our analyses. The GSE145996 (19) and phs000452.v2.p1 (22) datasets included 33 nonresponders and 23 responders of patients with melanoma. The GSE135222 (20) and GSE126044 (21) datasets included 30 nonresponders and 13 responders of patients with non–small cell lung cancer (NSCLC). Cindy Yang and colleagues (23) dataset included tumor samples collected from The Investigator-initiated Phase II Study of Pembrolizumab Immunological Response Evaluation (INSPIRE) cohorts of patients with advanced solid cancer treated with pembrolizumab, which included head and neck squamous cell carcinoma (HNSCC), triple-negative breast cancer, high-grade serous carcinoma, melanoma, and other mixed solid tumors. Using the classifications in Yang’s study, 18 pembrolizumab high-sensitivity/clinical benefit (responder) and 22 low-sensitivity (nonresponder) samples were analyzed in our study.

### Trial oversight

All biospecimens and data were collected as part of the single-center, observational study called Molecular Mechanisms of Tumor Evolution and Resistance to Therapy (MMTERT; ref. 49), which was approved by the Oregon Health & Science University (OHSU) Institutional Review Board (IRB #16113). Subject selection criteria included any patient over the age of 12 years with a suspected or confirmed diagnosis of localized, advanced, or metastatic cancer who is willing to undergo a biopsy. Both male and female participants were enrolled, and sex was recorded during data collection. Written informed consent was obtained from all participants prior to enrollment. The study was conducted in accordance with the ethical principles outlined in the Declaration of Helsinki and adhered to the International Council for Harmonization guidelines on Good Clinical Practice, in compliance with all applicable laws, regulatory requirements, and conditions mandated by regulatory authorities and institutional review boards. This study is part of the SMMART Clinical Trials Program at OHSU, which facilitates comprehensive molecular and spatial profiling of patient tumor samples to inform translational research.

### Clinical IHC

Tissue was formalin-fixed for ∼12 hours based on American Society of Clinical Oncology/College of American Pathologists guidelines (50). AR and CD4 IHC stains were performed in the OHSU Knight Diagnostic Laboratories on formalin-fixed, paraffin-embedded (FFPE) tissue, using prediluted antibodies intended for diagnostic use in a biotin-free protocol on a VENTANA BenchMark instrument. Staining was interpreted and scored by a pathologist. Antibodies were sourced from Roche and included anti-AR (Cell Marque, clone SP107), #760-4605, and anti-CD4 (CONFIRM, clone SP35), # 790-4423.

### NanoString GeoMx DSP

DSP was performed by the OHSU Knight Diagnostic Laboratories using a single FFPE slide per sample for protein panel analysis (NanoString GeoMx Digital Spatial Profiler; RRID: SCR_021660). FFPE sections (4–5 micron) were deparaffinized and subjected to antigen retrieval in citrate buffer, pH 6, for 15 minutes in a pressure cooker. Slides were then incubated at 4°C overnight with a cocktail of oligonucleotide-tagged antibodies along with two fluorescent-labeled morphologic markers: pan-cytokeratin (Alexa 532 nm) and CD45 (Alexa 594 nm). After antibody incubation, slides were blocked for 1 hour with Buffer W, washed with Tris-Buffered Saline with Tween 20 (TBS-T), and post-fixed with 10% neutral buffered formalin for 30 minutes. Following a brief wash with TBS-T, sections were incubated with SYTO-13 (FITC 525 nm) for 30 minutes and then placed on the DSP instrument for sample collection. Slides were scanned and tumor regions were identified based on nuclei staining and visualization markers. A minimum of three regions of interest (ROI; 660 micron diameter) were selected for each sample under the guidance of a board-certified pathologist in accordance with recommended practices (51). ROIs were chosen to maximize tumor cell content and to avoid necrotic areas or sectioning artifacts. UV-released oligonucleotide barcodes were collected automatically into a 96-well plate, hybridized with GeoMx Hyb Codesets at 67°C for 16 hours, and quantitated using the MAX nCounter system. A control tissue microarray was included in every run to correct for batch effects. Sample values were normalized by comparison with matching tumor-type reference cohorts, consisting of breast, ovarian, and sarcoma cancers. The normalized values were used for correlation analysis.

### Statistical analysis

All data processing, statistical analyses, and plotting were conducted in the R statistical computing environment software v4.2.0 (https://www.R-project.org/). Cox regression analyses were performed via the survival R package (version 3.3-1; refs. 52, 53). We calculated the correlation between two continuous variables using the Pearson correlation coefficients. The threshold of *P* < 0.05 (Pearson correlation test) indicates the significance of correlation. Wilcoxon rank-sum tests were performed on continuous measures between two groups to examine differences in distributions. All statistical tests were two-sided, and a *P* value < 0.05 was considered as statistically significant.

### Ethics statement

All analyses using publicly available, deidentified datasets (bulk and single-cell RNA-seq and immunotherapy cohorts) were exempt from IRB approval. The DSP data were collected under the MMTERT study, approved by the OHSU IRB (IRB #16113), with written informed consent obtained from participants.

### Patient and public involvement

Patients or the public were not involved in the design, conduct, reporting, or dissemination of this research as the RNA-seq data were obtained exclusively from publicly available resources.

## Results

### AR activity profiles in human cancers

**Figure 1.**
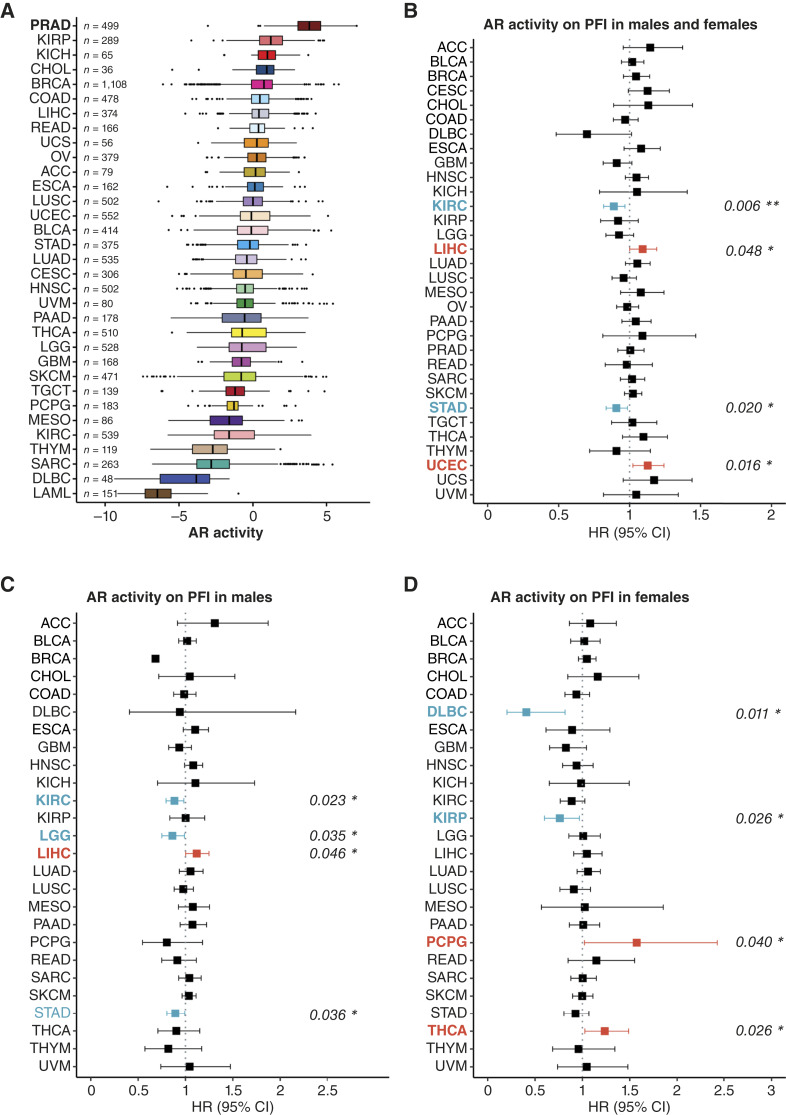
Overview of AR activity and association with PFI outcomes across 33 TCGA cohorts. **A,** Boxplot displaying AR activity ranked in order of decreasing median AR activity among 33 TCGA cancer types. Values on the left correspond to the total number of tumor samples (*n*) analyzed in each cancer cohort. TCGA study abbreviations and sample sizes for each dataset are listed in Supplementary Table S1. The center line indicates the median, the bounds of the box indicate the upper and lower quartiles, the whiskers indicate the minimum and maximum, and outliers are marked with dots. **B–D,** Univariate Cox regression analysis of AR activity on PFI endpoints was performed across TCGA cohorts, including pooled males and females (**B**), males only (**C**), and females only (**D**). No PFI data are available for LAML. **C** and **D,** include only cancer types with both male and female tumor samples. Note that in **C**, BRCA is not shown with the 95% CI because of the unreliability of the results caused by the small sample size. Forest plots display HR estimates, 95% CIs, and corresponding *P* values. Cancers for which AR activity significantly correlates with a favorable prognosis are highlighted in dark cyan, whereas those significantly associated with a poorer prognosis are highlighted in red-orange. Statistical significance: *, *P *< 0.05; **, *P* < 0.01; ***, *P* < 0.001.

To investigate AR signaling activity in human malignancies, instead of using the expression levels of individual genes, we utilized a regulon-based analysis. This approach is especially useful when investigating genes with low expression regulated by master regulators for which expression level is also unknown. Thus, we calculated AR activity using the VIPER algorithm (31), which employs the transcriptional regulatory network, including TFs and their targeted genes, to infer TF activity. Such a regulon-based method is more robust to noise and provides greater sensitivity in characterizing TF activities compared with measuring the gene expression of the TF itself (31). Figure 1A depicts the AR activity across all TCGA tumors (10,340 samples in 33 TCGA studies). These 33 cancers exhibited significant differences in the median AR activity (Kruskal–Wallis test; *P* < 2.2e−16), with each cancer type displaying a range of activity. Perhaps not surprisingly, because of its androgen dependency, prostate adenocarcinoma (PRAD) has the highest AR activity on average whereas LAML has the lowest (Fig. 1A). TCGA study abbreviations, sample sizes, and survival analysis data for each dataset are summarized in Supplementary Table S1 and Supplementary Data S2.

### The association between AR activity levels and patient survival outcomes

To gain insights into the clinical relevance of AR activity at the pan-cancer level, we evaluated the correlation between AR activity and PFI (Fig. 1B–D; Supplementary Data S2) and OS in TCGA cohorts (Supplementary Fig. S1; Supplementary Data S3). Tumor subtype analysis indicated that high AR activity is associated with a decreased risk for kidney renal clear cell carcinoma [KIRC; HR = 0.88; 95% confidence interval (CI), 0.82–0.97; *P* = 0.006; Fig. 1B] and stomach adenocarcinoma (STAD; HR = 0.91; 95% CI, 0.83–0.98; *P* = 0.020; Fig. 1B) and an increased risk for liver hepatocellular carcinoma (LIHC; HR = 1.09; 95% CI, 1.00–1.19; *P* = 0.048; Fig. 1B). The remaining cancer types did not exhibit significant differences in the relationship between AR activity and PFI outcomes of patients with cancer. Next, we asked how patient sex (as reported in TCGA) is associated with PFI. When separating patients by sex, high AR activity showed a positive association with survival in male patients with KIRC (HR = 0.88; 95% CI, 0.79–0.98; *P* = 0.023; Fig. 1C), STAD (HR = 0.89; 95% CI, 0.80–0.99; *P* = 0.036; Fig. 1C), and brain lower-grade glioma (LGG; HR = 0.86; 95% CI, 0.75–0.99; *P* = 0.035; Fig. 1C) and a negative association with LIHC (HR = 1.12; 95% CI, 1.00–1.25; *P* = 0.046; Fig. 1C). In contrast, high AR activity in female patients showed a positive association with survival in lymphoid neoplasm diffuse large B-cell lymphoma (DLBC; HR = 0.41; 95% CI, 0.20–0.82; *P* = 0.011; Fig. 1D) and kidney renal papillary cell carcinoma (KIRP; HR = 0.76; 95% CI, 0.60–0.97; *P* = 0.026; Fig. 1D) and a negative association with uterine corpus endometrial carcinoma (UCEC; HR = 1.13; 95% CI, 1.02–1.24; *P* = 0.016; Fig. 1B), thyroid carcinoma (THCA; HR = 1.24; 95% CI, 1.03–1.49; *P* = 0.026; Fig. 1D), and PCPG (HR = 1.57; 95% CI, 1.02–2.42; *P* = 0.040; Fig. 1D). Considering the roles of other sex hormone receptors such as ERs and PR in cancer progression, we also evaluated their activity in relation to PFI (Supplementary Fig. S2–S5; Supplementary Data S4–S6). Given previous evidence that a high AR/ER expression ratio is associated with poor prognosis in ER-positive breast cancer (54), we examined the AR/ERα activity ratio in males and females (Supplementary Fig. S3; Supplementary Data S4). In males, a higher AR/ERα ratio was associated with shorter PFI in KICH (HR = 2.08; 95% CI, 1.05–4.12; *P* = 0.036) and STAD (HR = 1.11; 95% CI, 1.02–1.20; *P* = 0.017), suggesting that AR dominance over ERα may worsen outcomes. Although LGG (HR = 1.00; 95% CI, 1.00–1.00; *P* = 0.039), PRAD (HR = 0.93; 95% CI, 0.88–0.98; *P* = 0.008), and KIRP (HR = 0.97; 95% CI, 0.94–1.00; *P* = 0.040) also reached statistical significance, their HRs were close to 1, indicating minimal biological impact. Interestingly, in male STAD, a high AR/ERα ratio increased risk, highlighting that the protective effect of AR may be reduced when ERα activity is relatively low. In females, the AR/ERα ratio showed significant associations with PFI in a few cancers although most HRs were close to 1 or derived from small sample sizes (Supplementary Fig. S3; Supplementary Data S4). In bladder urothelial carcinoma and THCA, the AR/ERα ratio showed modest effects associated with worse survival (HR = 1.12; 95% CI, 1.02–1.23; *P* = 0.018 and HR = 1.11; 95% CI, 1.01–1.23; *P* = 0.027, respectively). Notably, in female PCPG, higher AR activity alone was linked to poorer survival (Fig. 1D), whereas increased ERα activity tended to predict better outcomes (Supplementary Fig. S2D); however, a higher AR/ERα ratio was instead associated with improved survival, suggesting that relative balance rather than single-pathway dominance may be beneficial. These results underscore sex- and cancer-specific differences in the prognostic relevance of AR–ERα signaling as captured by transcriptome-based activity scores. However, in female breast cancer (BRCA), no significant association was observed (HR = 1.00; 95% CI, 0.98–1.02; *P* = 0.652), in contrast to previous IHC-based studies in ER-positive breast cancer, reflecting the biological heterogeneity of the TCGA-BRCA cohort.

### AR activity–correlated genes are significantly associated with immune system processes

**Figure 2.**
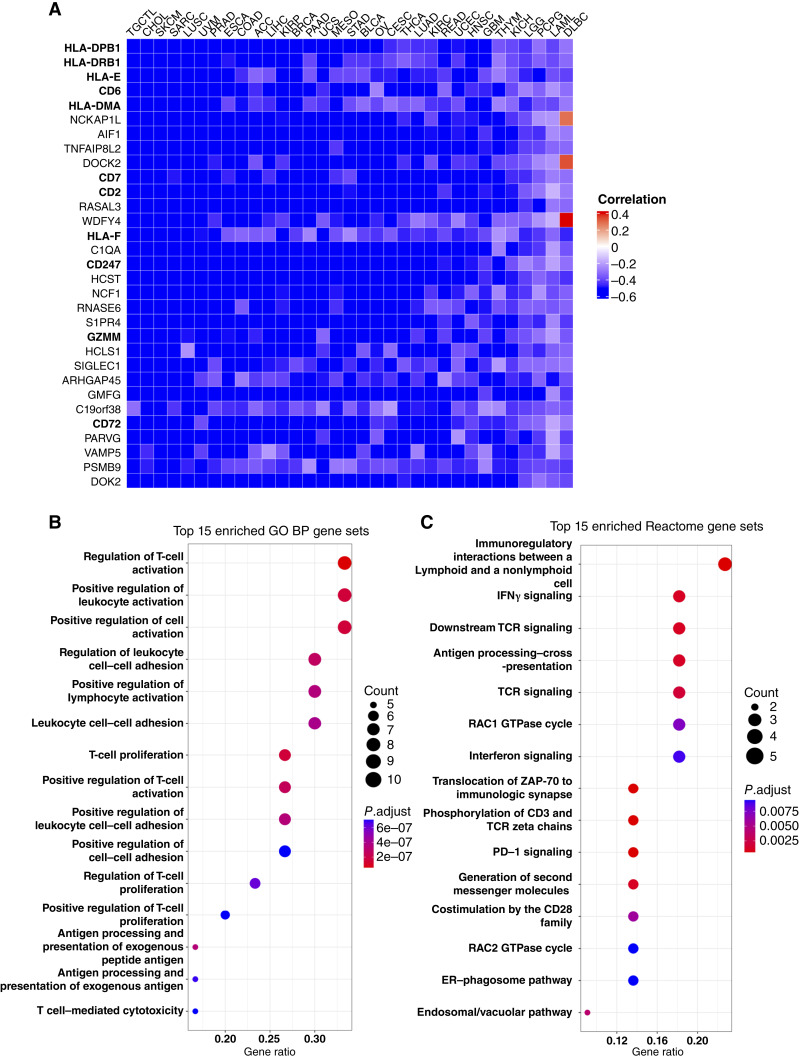
AR activity shows a strong negative correlation with genes involved in immune system processes. **A,** Evaluation of the correlation between AR activity and each gene expression within the entire dataset of each tumor type across all 33 TCGA cohorts. Heatmap displaying the 31 genes (rows) that are significantly correlated with AR activity across all 33 tumor types. Pearson correlation tests were used to calculate the correlation between AR activity and each gene’s expression in each cancer type. Significant correlations (*P* < 0.05) are indicated in red (positive correlation) or blue (negative correlation). Cancer types (columns) are sorted by decreasing mean correlation coefficient. **B** and **C,** Pathway enrichment analysis of the 31 AR activity–correlated genes. Dot plots indicating the top 15 significantly enriched GO BP terms (**B**) and Reactome pathways (**C**). The vertical axes represent the function classifications or pathways, and the horizontal axes represent the ratio of genes contained in the ontology or pathways. The dot’s color indicates the adjusted *P* value for enrichment significance, and the size of the dot indicates the number of genes in the functional class or pathway.

Based on our previous work demonstrating that AR can repress antitumor T-cell function (11) together with our observations of AR activity and the association with PFI and/or OS, we sought to investigate the potential impact of AR activity on gene expression. We performed a genome-wide gene expression correlation experiment and calculated the Pearson correlation coefficients to assess the relationship between AR activity and the expression of each gene in each cancer type. Genes significantly correlated with AR activity were identified by *P* < 0.05. We identified 31 genes significantly associated with AR activity across 33 cancer types (Fig. 2A). Remarkably, of the 1,023 correlation coefficients calculated between these genes and AR activity, 99.7% were negative (1,020 negative correlations and three positive correlations). Among the negative correlations, 632 were strongly negative, with coefficients less than −0.5. Notably, several MHC class I and II genes (*HLA-E*, *HLA-F*, *HLA-DPB1*, *HLA-DRB1*, and *HLA-DMA*), as well as T cell– and B cell–specific genes (*CD2*, *CD6*, *CD7*, *CD247*, and *CD72*), were among the 31 genes most frequently and negatively correlated with AR activity (Fig. 2A). Additionally, we used GO BP and Reactome pathway analysis to gain further insights into the underlying biology of these 31 genes. These genes were predominantly involved in immune processes such as T-cell activation regulation, leukocyte activation, leukocyte cell–cell adhesion, T-cell proliferation, antigen processing and presentation, T-cell receptor signaling, and interferon signaling (Fig. 2B and C). Such unbiased analyses revealed that AR activity is negatively correlated with genes involved in positive immune processes, suggesting immunosuppressive effects of AR in multiple cancer subtypes.

### AR activity negatively correlates with immune infiltration across cancers

**Figure 3.**
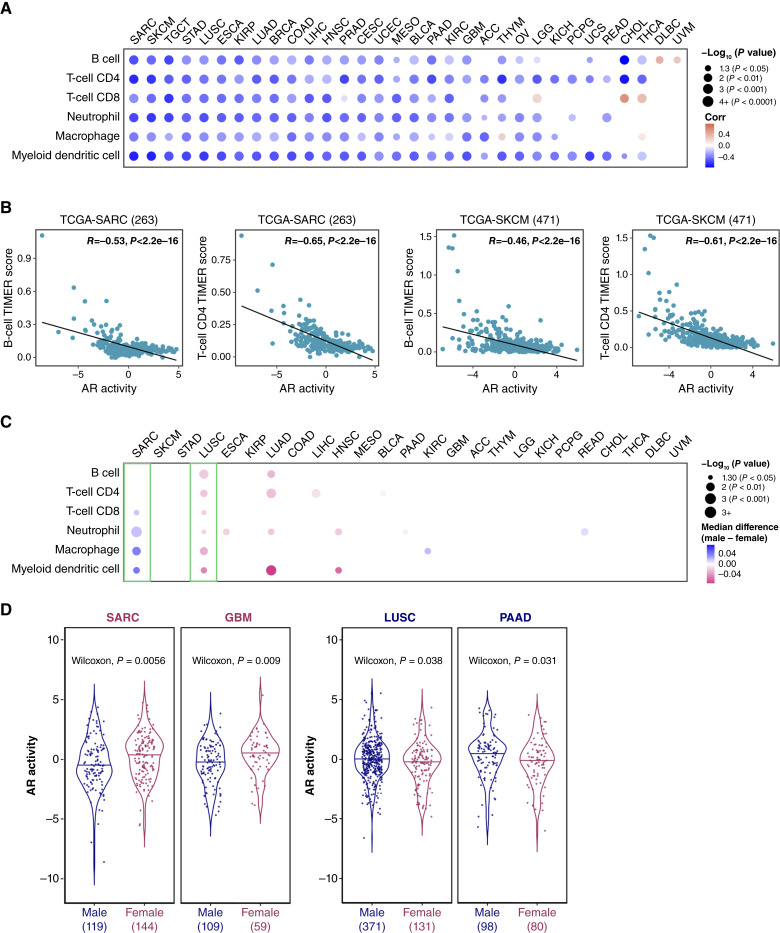
AR activity exhibits a negative correlation with immune infiltration across cancers. **A,** Correlations between AR activity and six immune cell populations (B cells, CD4^+^ T cells, CD8^+^ T cells, macrophages, dendritic cells, and neutrophils) across 32 cancer types (LAML is not applicable in TIMER calculation). Each circle represents a correlation coefficient value analyzed by a two-tailed Pearson correlation test. Positive correlation coefficients are displayed in orange and negative correlation coefficients in blue. The color intensity is proportional to the correlation coefficients. The circle size corresponds to the *P* values, with correlation coefficients having *P* values > 0.05 left blank. Cancer types (columns) are sorted in order of decreasing mean of correlation coefficient. The enrichment scores of immune infiltration levels are determined by the TIMER algorithm. **B,** Representative scatter plots demonstrating the dots in the top left of (**A**) that show the correlation between AR activity and B cells or CD4^+^ T cells in SARC and SKCM cohorts. **C,** Differences in tumor immune infiltration levels between male and female patients in 25 TCGA cohorts. The dots indicate significant median differences in tumor immune infiltration levels, with *P* values > 0.05 left blank. Blue and pink dots represent higher immune infiltration levels in males and females, respectively. The color intensity is proportional to the number of median differences. The circle size corresponds to the *P* values calculated using a two-sided Wilcoxon rank-sum test. Cancer types (columns) are sorted in accordance with **A**. **D,** Violin plots illustrating AR activity differences in four TCGA cohorts between male (navy) and female (maroon) patients. Statistical significance was determined using two-sided Wilcoxon rank-sum tests between males and females. The values at the bottom indicate the total number of tumor samples in each condition.

Given our observation that AR activity is negatively associated with expression levels of immune-related genes, we investigated the correlation between AR activity and immune cell composition in tumors. We utilized the TIMER analysis, which is an immune deconvolution method, to estimate the abundance of immune cell composition in tumors (38). Similarly, we found that AR activity negatively correlates with six leukocyte populations, including B cells, CD4^+^ T cells, CD8^+^ T cells, neutrophils, macrophages, and myeloid dendritic cells (Fig. 3A). Among 32 TCGA cancer types (LAML is not applicable in TIMER calculation), AR activity shows moderate-to-strong negative correlations (correlation coefficients ranging from −0.4 to −0.8) with the abundance of these six immune cell types in 19 cancers as estimated by TIMER. Particularly, sarcoma (SARC) and skin cutaneous melanoma (SKCM) exhibited the highest degree of negative correlations between AR activity and immune infiltration (Fig. 3A and B). Beyond the six immune cell types estimated by TIMER, we also conducted ssGSEA to quantify the degree of infiltration of 28 human immune cell types (39) in each bulk RNA-seq sample and computed its correlation with AR activity (Supplementary Fig. S6). Once again, AR activity showed a negative correlation with immune cell infiltration across cancer lineages. Moreover, recognizing sex as a biological variable that influences various immune system functions (55), we assessed differences in immune infiltration between male and female patients (Fig. 3C). Among 25 cancer types with sample sizes greater than 12 per male or female group, we performed the Wilcoxon test for statistical analysis. Four cancer types [SARC, lung squamous cell carcinoma (LUSC), lung adenocarcinoma (LUAD), and HNSCC] showed differences in the abundance of more than two of six immune cell types (Fig. 3C) between male and female tumors. Male patients with SARC showed higher infiltrating levels of CD8^+^ T cells, neutrophils, macrophages, and myeloid dendritic cells whereas female patients with LUSC and LUAD had higher infiltration levels of multiple immune cell types (Fig. 3C). We also investigated the correlation between ERs, PR activity, and immune infiltration in tumors (Supplementary Fig. S7). Unlike AR, ERs and PR showed positive correlations with immune infiltrates in most cancer types. Next, we examined the differences in AR, ER, and PR activity between sexes for cancer types with at least 12 samples from both females and males (Fig. 3D; Supplementary Fig. S8). AR activity was higher in male tumors in LUSC and pancreatic adenocarcinoma [Fig. 3D (right); *P* < 0.05], whereas AR activity was higher in female tumors in glioblastoma multiforme and SARC [Fig. 3D (left); *P* < 0.05]. Notably, female patients with SARC and male patients with LUSC exhibited higher AR activity (Fig. 3D) and lower immune infiltrates (Fig. 3C), suggesting that AR signaling activation may contribute to reduced immune infiltration observation in these populations. We further evaluated the association between AR activity and immune cell abundances across all tumor types in TCGA, stratified by sex (Supplementary Fig. S9). Taken together, AR activity was negatively correlated with immune infiltration in both males and females.

### AR activity negatively correlates with prognostic immune signatures across cancers

**Figure 4.**
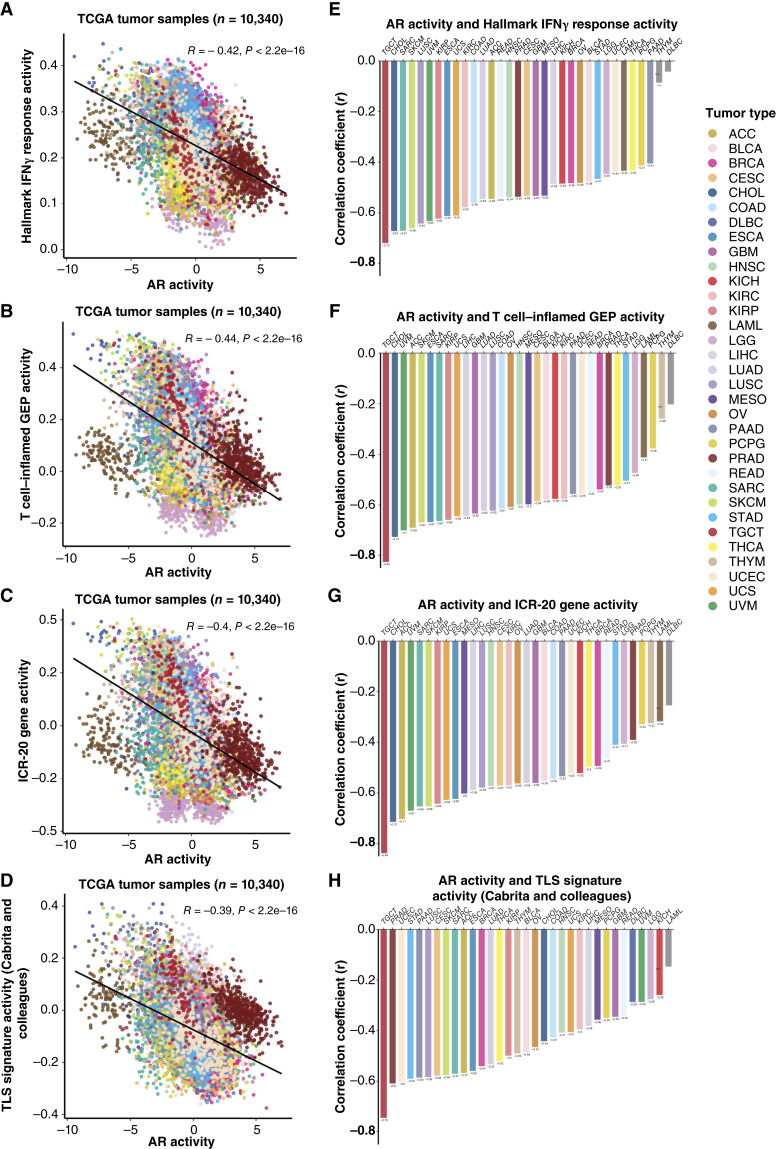
AR activity levels are negatively correlated with prognostic gene signatures across 33 TCGA cohorts. Scatter plots (**A–D**) showing the Pearson correlation of AR activity with (**A**) Hallmark IFNγ pathway; (**B**) T cell–inflamed GEP; (**C**) ICR-20 gene; and (**D**) TLS signature activity scores of all TCGA tumor samples. Each dot represents one tumor sample (*n* = 10,340), with colors indicating different tumor types. Tumor types are listed on the right by color code (*n* = 33). Bar plots (**E–H**) displaying the Pearson correlation of AR and signature activities within each cancer type. The *X*-axis denotes cancer types arranged in decreasing correlation coefficient value (*Y*-axis). The texts below each bar indicate the Pearson correlation coefficients. Bars on the far right with *P* values > 0.05 are colored in light gray. ns, nonsignificant.

To further examine whether AR activity is associated with immunologic activity in the TME, we investigated three IFNγ-related immune gene expression signatures and one signature of TLS known to be associated with favorable responses to ICIs and of prognostic value. These included the Hallmark IFNγ signaling (41), the T cell–inflamed gene GEP (42), the ICR-20 gene signature (43), and the TLS signature (full gene list in Supplementary Data S1; a Venn diagram of the signatures is provided in Supplementary Fig. S10; ref. 44). The T cell–inflamed GEP has been evaluated and shows predictive efficacy in patients treated with pembrolizumab across 20 cancers in the clinical trial KEYNOTE-028 (56). The ICR-20 gene signature was independently associated with improved outcomes from anti–PD-1/–PD-L1) ICIs in patients with advanced NSCLC (57). We performed ssGSEA to calculate immune signature scores in the TCGA bulk RNA-seq dataset and analyzed the correlation between AR activity and the multigene immune signature scores. Significant negative correlations were observed between each signature score and AR activity across all TCGA tumor samples (*n* = 10,340), with Pearson correlation values ranging from −0.39 to −0.44 (Fig. 4A–D; Supplementary Fig. S11). This observation was unique to AR as similar trends were not observed with ERs or PR (Supplementary Fig. S12–S14). The correlations between AR activity and each immune signature score within each cancer type are displayed in Fig. 4E–H. In most cancers, except DLBC and Thymoma, robust and significant inverse relationships concordantly existed between AR activity and the prognostic immune signatures. Strong negative correlations were observed in approximately one third of cancers (Pearson correlation < −0.6; *P* < 2.2e−16). These results suggest that AR signaling is negatively associated with tumor immunity across cancers. To determine whether this observation was unique to the tumor or shared by the tissue of origin, we also explored the correlation between AR activity and the signature activities in normal tissues from the GTEx database (Supplementary Table S2; Supplementary Fig. S15–S17; ref. 8). Once again, AR activity exhibited significant negative correlations with these immune signature activities in GTEx samples, suggesting conserved AR regulation of immunity in healthy tissue and tumor.

### Inhibiting AR signaling increases immune infiltration and IFNγ signaling activity in patients with mCRPC

**Figure 5.**
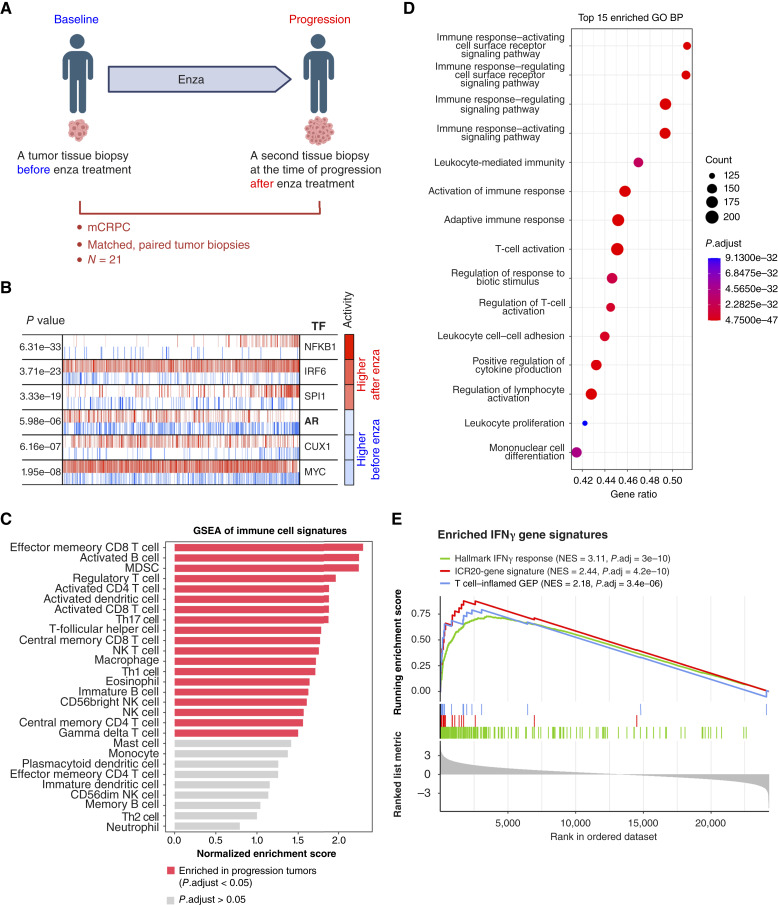
Master regulator and pathway enrichment analyses reveal decreased AR activity, increased immune cell signature activity, and enrichment of the IFNγ response after enza treatment in 21 matched-biopsy patients with mCRPC. **A,** Study schematic. **B,** msVIPER plot depicting the top three TFs predicted to be most activated (red in activity) or deactivated (blue) in progression tumor samples compared with baseline tumor samples. Tick marks in red or blue lines represent targets of TFs that are positively or negatively regulated, respectively. **C,** GSEA of 28 immune cell signatures indicating immune infiltration in progressing tumor samples. **D,** GSEA of GO BP demonstrating the top 15 enriched pathways (with normalized enrichment score ranging from 2.71 to 2.82) in progression tumor samples associated with immune system processes. **E,** GSEA plot showing significantly activated three prognostic gene signatures after enza treatment. Adj., adjusted; NES, normalized enrichment score. (**A,** Created in BioRender. Xia, Z. (2025) https://BioRender.com/vnzdcsz.)

Previous studies in patients with primary prostate cancer have revealed that androgen axis blockade increases immune infiltrates in the prostate (58–60). Despite this evidence, advanced prostate cancer remains relatively devoid of immune infiltrates (61). Given the use of second-generation AR inhibitors in advanced prostate cancer, we sought to understand whether these agents modify the immune landscape of mCRPC. Therefore, we conducted RNA expression profiling of metastatic tumor tissues from matched patient biopsies before and after treatment with the AR signaling inhibitor enza, one of the principal treatments for mCRPC (Fig. 5A; ref. 62). Indeed, master regulator analysis of the two conditions identified that AR activity was reduced in tumor samples after treatment (Fig. 5B), confirming that enza inhibited AR activity in these patients. Consequently, GSEA indicated that, following treatment, tumor samples showed increased gene expression associated with immune cell signaling in 19 of 28 immune cell types (Fig. 5C). Additionally, there was significant upregulation of genes involved in various positive immune process pathways as reflected by GO BP terms (Fig. 5D). Moreover, GSEA results demonstrated significant enrichments of prognostic immune signatures in tumor samples after AR signaling inhibition by enza treatment, including the Hallmark IFNγ signaling signature, the 18-gene T cell–inflamed GEP, and ICR 20-gene signatures (Fig. 5E). These observations suggest that AR inhibition leads to increased immune cell infiltration and could potentially enhance the response to immune checkpoint blockade (ICB) in tumor types beyond prostate cancer.

### AR activity level correlates inversely with response to ICB treatment

**Figure 6.**
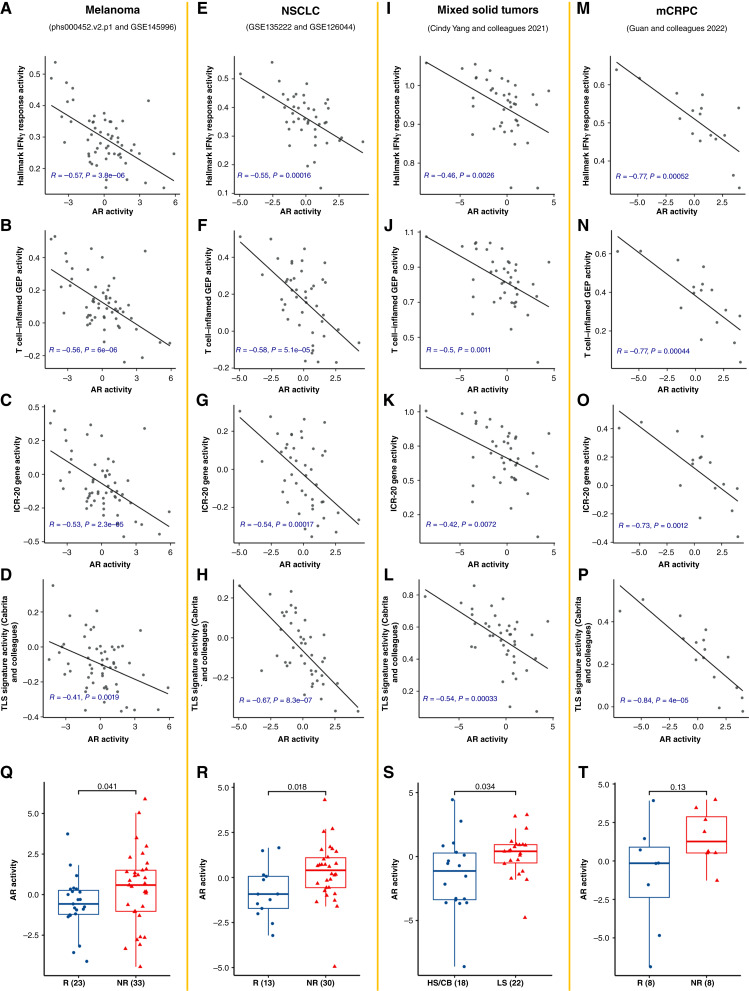
AR activity level inversely correlates with response to ICB treatment in public datasets. **A–P,** Scatter plots showing Pearson correlations of AR activity and the Hallmark IFNγ pathway, T cell–inflamed GEP, ICR-20, and TLS gene signature activity scores for the phs000452.v2.p1 and GSE145996 combined melanoma dataset (**A–D**), the GSE135222 and GSE126044 combined NSCLC dataset (**E–H**), Yang and colleagues mixed tumors dataset (**I–L**), and the Guan and colleagues prostate cancer dataset (**M–P**). **Q–T,** Boxplots showing AR activity levels between responders and nonresponders to ICB treatment in the melanoma dataset (**Q**, *P* = 0.041), the NSCLC dataset (**R**, *P* = 0.018), and Guan and colleagues prostate cancer dataset (**T**, *P* = 0.13), as well as the high-sensitivity/clinical benefit (HS/CB) and low-sensitivity (LS) groups in mixed tumors dataset (**S**, *P* = 0.034). NR, nonresponder; R, responder.

Because the immune landscape of the TME is associated with prognostic outcomes and because of the observed inverse relationships between AR activity and immune signatures, we hypothesized that AR activity in patient baseline tumors might correlate with their response to immunotherapy. To test this hypothesis, we first investigated the correlation between AR activity and the four prognostic immune signatures (41–44, 56) using six publicly available RNA-seq datasets from pretreatment tumor samples that include response data after ICB treatment from various cancer types [GSE145996 (19), phs000452.v2.p1 (22), GSE135222 (20), GSE126044 (21), Cindy Yang and colleagues (23), and Guan and colleagues (11); Fig. 6]. A strong negative correlation (Pearson correlation < −0.5) was observed in almost all datasets between AR activity and the immune signatures (Fig. 6A–P). Subsequently, we compared AR activity levels between nonresponders and responders in these cancer cohorts. We found that responders exhibited a significantly lower AR activity level than nonresponders in melanoma, NSCLC, and mixed tumor cohorts, respectively (*P* < 0.05, two-sided Wilcoxon rank-sum test; Fig. 6Q–S). Of note, responders had a trend toward lower AR activity in patients with mCRPC (*P* = 0.13; Fig. 6T). Our results indicated that AR signaling negatively correlates with the response of patients with cancer to ICB. This suggests that the level of AR activity in tumors prior to ICB treatment may provide insights into preexisting tumor immune microenvironment and influence therapy selection.

### AR activity is inversely correlated with immune infiltration from single-cell analysis

To further investigate the relationship between tumor cell–intrinsic AR signaling and immune infiltration at single-cell resolution, we utilized an androgen-dependent tumor model, prostate cancer, and an associated comprehensive scRNA-seq atlas of human prostate cancer (Supplementary Fig. S18A and S18B; tumor samples = 29 and total cells = 79,830; ref. 28). The results showed a significant negative correlation between AR activity in the epithelial compartment and the level of immune cell infiltration in the TME of human prostate cancer (Supplementary Fig. S18C). Additionally, by reanalyzing the recently published human prostate cancer scRNA-seq dataset (total samples = 12 and total cells = 31,231; ref. 30), we observed a significant increase in immune cell infiltration within the prostate cancer TME following ADT (Supplementary Fig. S18D). Taken together, these findings suggest that tumor cell–intrinsic AR signaling may have an immunosuppressive effect within the human prostate cancer TME.

### AR protein expression correlates inversely with CD45 and CD4 in the TME

**Figure 7.**
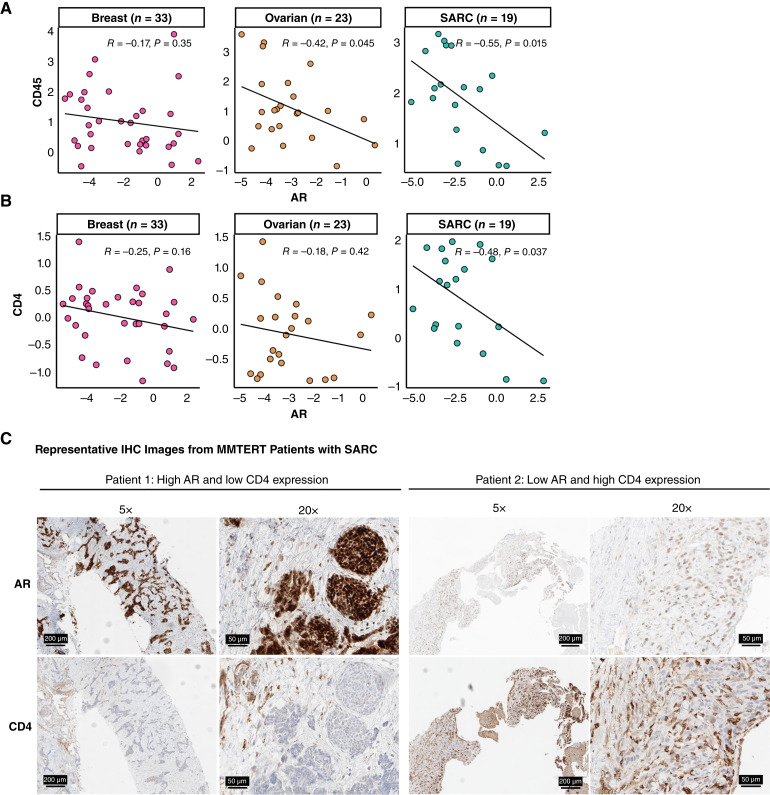
Correlation analysis of AR protein expression with immune cell markers CD45 and CD4 in DSP protein profiling across breast, ovarian, and sarcoma cancer types. Scatter plots show the correlation between AR and (**A**) CD45 protein expression and (**B**) CD4 protein expression. Protein expression values were normalized to the standard control probe. **C,** Representative IHC images of AR and CD4 expression in tumor tissues from two patients with sarcoma. For each marker, the left panels show images at 5× magnification and the right panels show images at 20× magnification. Brown staining indicates positive expression.

Our pan-cancer analysis revealed a strong negative correlation between AR activity and immune infiltration in both bulk RNA-seq and single-cell transcriptomic data. We also observed that AR activity varied not only across cancer types but also among patients within each cancer cohort. These findings prompted us to investigate whether similar patterns could be detected at the protein level. To this end, we leveraged a curated DSP protein dataset from the MMTERT trial conducted at Knight Cancer Institute at OHSU, which includes samples from breast, ovarian, and SARC cancers. Our aim was to assess the relationship between AR protein expression and immune cell markers, specifically CD45 and CD4, within the TME. Notably, AR expression showed a moderate negative correlation with CD45, a general marker of leukocytes, in both patients with ovarian cancer and SARC (Fig. 7A). Similarly, a moderate negative correlation was observed between AR and CD4, a marker of helper T cells, in patients with SARC (Fig. 7B). In contrast, no correlation was found between ERα, PR, and immune markers (Supplementary Fig. S19A–S19D). IHC staining of AR and CD4 in SARC tumor tissues revealed that tumors with high AR expression exhibited low CD4 infiltration [Fig. 7C (left)], whereas tumors with low AR expression showed high CD4 infiltration [Fig. 7C (right)]. These findings provide compelling evidence at the protein level of a potential link between AR signaling and tumor-infiltrating leukocyte abundance in these cancers.

## Discussion

AR has been implicated as a suppressor of immune function in melanoma, prostate, and bladder cancer (10, 11, 13–15, 63). In this study, we applied a network computational approach to investigate AR, ER, and PR activity in human cancers using 33 bulk tumor RNA-seq datasets from TCGA cohorts. We provide a comprehensive view of the association between AR activity and patient survival, associated genes, and immune cell infiltration. Our analysis revealed clear negative correlations of pan-cancer AR activity with tumor immune infiltrates in both sexes. We also demonstrated that AR inhibition correlates with enriched immune cell signaling pathways in advanced prostate tumors, using a unique collection of matched biopsies from patients with prostate cancer taken before treatment with enza and upon progression. Consistent with these transcriptomic findings, analysis of DSP protein data further showed that AR expression inversely correlates with immune markers CD45 and CD4 in the TME. Notably, our findings suggested that low AR activity may reflect higher immune infiltrates and IFNγ immune signature activity in the TME, which is also associated with improved response to ICB treatment in various cancers.

The role of AR signaling in cancer development is complex and multifaceted. AR can be activated in ligand-dependent and -independent manners (64). In the ligand-independent mechanism, signaling is independent of AR binding DNA but rather through association with cytoplasmic proteins, which occurs relatively often in cancer (65). In fact, AR signaling has been shown to interact with several oncogenic signaling pathways involved in promoting growth and resistance across various tumor types, such as PI3K/AKT/mTOR, EGFR, HER2/Neu, and Wingless-related integration site (65–69). Moreover, AR signaling may interplay with other hormone receptors such as ER and glucocorticoid receptor, which can modulate transcriptional programs and potentially influence the response to AR-targeted therapies (54, 70, 71). This cross-talk may be particularly relevant in hormone-dependent cancers such as breast and prostate cancers, in which inhibition of the dominant hormone receptor (i.e., AR in prostate cancer) can lead to alternative hormone receptors supporting tumor cell growth by hijacking the signaling cascade. Nevertheless, studies correlating AR expression with clinical outcomes have not been consistent. Perhaps unsurprising is that our analysis showed that PRAD has the highest levels of AR activity compared with other tumors, reflecting the tumor dependence on androgens. In contrast, higher AR activity is associated with better survival in KIRC. These findings align with previous research in renal cell carcinoma, which demonstrated that AR-positive tumors exhibit a more favorable prognosis (72, 73). Similar conclusions from previous research suggest that AR protein expression is significantly associated with low-stage, well-differentiated tumors and a favorable prognosis in patients with clear cell renal cell carcinoma (74–76). In contrast, two other studies found that increased expression of *AR* mRNA was associated with poor prognosis in renal cell carcinoma (77, 78). Clearly, a more rigorous investigation of the nuances of AR biology in kidney cancer is needed to understand the clinical significance of high versus low AR activity in this disease.

Aside from kidney cancer, our results reveal that high AR activity is associated with poor survival in patients with UCEC and SKCM (OS endpoint). UCEC is a hormone-sensitive disease, influenced by both estrogen and progesterone (79). A previous study demonstrated that a significant proportion of metastatic endometrial cancers express AR (80). Additionally, AR is more frequently expressed in metastatic lesions than ERα and PRs, indicating that AR could be a potential therapeutic target, particularly in patients with a high AR-to-ERα ratio (80). In regard to SKCM, others have reported that melanoma cells rely on sustained AR signaling for proliferation and tumorigenesis, and downregulation correlated with enhanced immune cell infiltration intratumorally in both sexes (63), which is consistent with the findings in the current study. Furthermore, we and others have demonstrated that BRAF-/MEK-targeted therapies for the treatment of advanced melanoma induce AR expression in males and females; however, treatment outcomes are worse in male patients (81).

It is well known that males are more susceptible to several cancers of nonreproductive tissues (82), yet the genetic and/or cellular reasons for such differences are poorly understood. In this study, we investigated the relationship between AR activity and immune cell signaling across many cancer types. This analysis revealed some interesting observations that highlight the nuances of hormone receptor biology in different tumor types. For example, we discovered that in LUSC, AR activity was higher in male tumors. In contrast, in SARC, female tumors exhibited higher AR activity. Despite these sex differences, both tumor types retained an inverse relationship between AR signaling and immune infiltration. At the protein level, nuclear AR has been detected in 70% to 98% of nonsquamous salivary duct carcinoma (SDC) cases (83), with a higher prevalence observed in men (84). Among patients with SDC, those who received first-line ADT showed a better response rate and comparable survival outcomes compared with those who underwent first-line chemotherapy (85). It has also been reported that AR-mediated T-cell exhaustion was more pronounced in male T cells than in female T cells in melanoma (14) and bladder cancer (13). Our results extend these observations and suggest that not only are there differences in T-cell function, but AR activity is negatively correlated with genes involved in active immune system processes.

Leveraging a unique dataset with matched biopsies before and after AR inhibition with enza treatment, we identified enriched immune cell signaling pathways and IFNγ signaling activity in the patients’ on-treatment tumor samples that showed AR deactivation. Consistent with these findings, a recent study in patients with ER^+^/HER2^−^ breast cancer treated with neoadjuvant fulvestrant with or without enza demonstrated that AR inhibition increased expression of immune activation gene sets, including IFNγ signaling, and reduced immunosuppressive myeloid populations (86). This suggests that the immune-modulatory effects of AR inhibition are not limited to prostate cancer but extend to other tumor types. Extending these observations, we found that AR activity is negatively associated with IFNγ pathway activity at the pan-cancer level. IFNγ, produced by activated T cells and NK cells within the TME, plays a central role in antitumor immunity (87). Tumors with higher IFNγ signaling gene expression are more likely to respond to ICB, such as anti–cytotoxic T-lymphocyte–associated protein 4 (CTLA-4) and anti–PD-1/–PD-L1 (42, 88–90).

To represent IFNγ signaling activity in tumor samples, we applied ssGSEA to compute signature scores for three established IFNγ-related signatures: the Hallmark IFNγ response and the T cell–inflamed GEP signatures, which include IFNγ-responsive genes involved in antigen presentation, cytotoxic activity, chemokine expression, and adaptive immune resistance (41, 42), and the ICR 20-gene signature, which reflects the strength of cytotoxic responses characterized by Th1 signaling, chemoattraction, cytotoxic function, and immune checkpoint–related genes (43, 90). Our results from healthy tissue (GTEx) and tumor (TCGA) analysis demonstrated a significant negative correlation between AR activity and the three IFNγ-associated signature scores in normal tissue and tumors, suggesting conserved AR-regulated biology irrespective of malignancy status. Importantly, a recent longitudinal study of patients receiving gender-affirming testosterone treatment suggests that testosterone represses many of the pathways and/or gene signatures that we observe to be negatively associated with elevated AR activity (7).

Our findings across cancer types suggest that deactivation of AR in the TME reflects higher IFNγ signaling activity, which may indicate greater responsiveness to ICB therapy. Indeed, we analyzed transcriptomic data from tumors collected prior to ICB treatment across six independent datasets encompassing multiple cancer types treated with anti–PD-1/–PD-L1 or anti–CTLA-4 therapies. We found that nonresponders exhibited higher AR activity than responders. These results extend our previous work demonstrating, in both preclinical and clinical contexts, that AR is a negative regulator of CD8^+^ T-cell responsiveness to anti–PD-1/–PD-L1 treatment (11). Our DSP analysis further supports this idea by revealing that AR protein expression negatively correlates with CD45 abundance in ovarian and SARC cancers, and with CD4 in SARC, reinforcing AR’s role in promoting an immunosuppressive TME. Collectively, these findings, including our recent study published in Cancer Discovery (2025; ref. 15), position AR not only as an oncogenic driver but also as a key mediator of immune evasion.

There are some limitations to this study. Our analyses primarily relied on data from the TCGA database, which consists largely of primary tumor samples. However, some TCGA samples may not be fully treatment naïve, and co-medication information was not available for TCGA or the other datasets analyzed, so these factors could not be accounted for. Additionally, the included studies involving ICB treatment did not encompass all cancer types. Future studies with larger sample sizes and broader cancer type representation, including subtypes, would help to further clarify the potential role of AR activity in predicting ICB response. Finally, our observations beg the question of why immunotherapy in advanced prostate cancer has limited clinical efficacy when standard of care is androgen pathway blockade (91). Herein, we make no attempt to answer this question and, rather, provide a thorough framework across multiple solid tumors that suggests that AR activity is inversely correlated with immune infiltration and may provide a novel biomarker for identifying tumors that would benefit from hormone axis inhibition with their immunotherapy. To this point, a recent phase I study in patients with PD-1–refractory melanoma treated with ADT showed a remarkable ability of AR inhibition to sensitize to immunotherapy (92).

In summary, this study reveals a robust negative association between AR activity and both tumor immune infiltration and immunotherapy response across multiple tumor types, independent of sex. These findings suggest that combining immunotherapies with AR blockade may represent a promising treatment strategy, particularly in tumors with elevated AR activity across various solid cancers, potentially reversing AR-driven immune suppression within the TME.

## Supplementary Material

Supplementary Data 1 Prognostic immune gene signatures

Supplementary Data 2 Data for Figure 1B to 1D

Supplementary Data 3 Data for Supplementary Figure S1

Supplementary Data 4 Data for Figure S2B to S2D and S3A, B

Supplementary Data 5 Data for Figure S4B to S4D

Supplementary Data 6 Data for Figure S5B to S5D

Supplementary Table S1 Abbreviations and evaluated samples in TCGA cohorts

Supplementary Table S2 Abbreviations and evaluated tissue sample numbers in the GTEx database.

Supplementary Figure S1 AR activity on overall survival outcome in pooled male and female samples across 33 TCGA cohorts.

Supplementary Figure S2 Overview of ERα activity and association with progression-free interval outcomes across TCGA cohorts.

Supplementary Figure S3 Association between AR/ESR1 ratio and progression-free interval (PFI) across TCGA cohorts.

Supplementary Figure S4 Overview of ERβ activity and association with progression-free interval outcomes across TCGA cohorts.

Supplementary Figure S5 Overview of PR activity and association with progression-free interval outcomes across TCGA cohorts.

Supplementary Figure S6 Correlation between AR activity and immune cell enrichment across 33 cancer types.

Supplementary Figure S7 Correlations between ERa, ERb, and PR activity with six immune cell populations in all tumor samples across 32 TCGA cancer types.

Supplementary Figure S8 Comparing nuclear receptor activity between males and females in 27 cancer types.

Supplementary Figure S9 Correlations between AR activity and six immune cell populations in males and females.

Supplementary Figure S10 Venn Diagram of Immune Signatures.

Supplementary Figure S11 Correlations between AR activity and immune signatures with overlapping genes removed.

Supplementary Figure S12 ERa activity levels are positively correlated with gene signatures of three prognostic gene signatures and TLS across 33 TCGA cohorts.

Supplementary Figure S13 ERb activity levels are positively correlated with gene signatures of three prognostic gene signatures and TLS across 33 TCGA cohorts.

Supplementary Figure S14 Correlation between PR activity and with gene signatures of three prognostic gene signatures and TLS across 33 TCGA cohorts.

Supplementary Figure S15 AR activity levels are negatively correlated with gene signatures of IFN-γ signaling and TLS in GTEx tissue samples.

Supplementary Figure S16 Overview of AR Activity across 54 GTEx tissue types. Boxplots displaying AR activity ranked in order of decreasing AR activity median among 54 GTEx tissue types.

Supplementary Figure S17 Correlations between activity and immune signature scores in tissue types from the GTEx database.

Supplementary Figure S18 AR activity levels are negatively correlated with immune cell infiltration in TME of human PCa evaluated by scRNA-seq.

Supplementary Figure S19 Correlation analysis of ERα and PR expression with immune cell markers CD45 (A, B) and CD4 (C, D) in Digital Spatial Profiling protein data across breast, ovarian, and sarcoma cancer types.

## Data Availability

The publicly available datasets used in this study can be accessed from multiple sources. The datasets from GDC (https://portal.gdc.cancer.gov/) are available via the TCGAbiolinks R/Bioconductor package (version 2.24.1; refs. 16–18), whereas the GEO (https://www.ncbi.nlm.nih.gov/geo/) database contains GSE145996 (19), GSE135222 (20), and GSE126044 (21), and dbGaP (https://www.ncbi.nlm.nih.gov/gap/; phs000452.v2.p1; ref. 22) is another source. Additionally, the source data from literatures such as Zhang and colleagues (28), Zheng and colleagues (29), Hawley and colleagues (30), Cindy Yang and colleagues (23), Westbrook and colleagues (24), and Guan and colleagues (11) were used in this study. Data generated in this study are available upon request from the corresponding author. All software packages used in this study are publicly available. No original code was developed. The analysis scripts and demo data are available in our Code Ocean capsule at https://codeocean.com/capsule/7694890/tree/v3. Additional information can be obtained from the corresponding author upon request.
