# Supplementary material for: Elevated Tumor-Associated Androgen Receptor Activity Correlates with Poor Immune Infiltration and Immunotherapy Response across Cancer Types
Source: Cancer Res Commun. 2026 Jan 5;6(1):17–35. doi: 10.1158/2767-9764.CRC-25-0409 (PMC12766373; doi:10.1158/2767-9764.CRC-25-0409)
Supplement: Supplementary Table S1 — Abbreviations and evaluated samples in TCGA cohorts [file crc-25-0409_supplementary_table_s1_suppst1.pdf]

Supplementary Table S1. Abbreviations and evaluated samples in TCGA cohorts

Symbol	Name	RNA-Seq Tumor Samples (n)	PFI* All samples (n)	PFI* Male (n)	PFI* Female (n)
ACC	Adrenocortical carcinoma	79	79	31	48
BLCA	Bladder urothelial carcinoma	414	408	301	107
BRCA	Breast invasive carcinoma	1108	1080	12	1068
CESC	Cervical squamous cell carcinoma and endocervical adenocarcinoma	306	303	NA	303
CHOL	Cholangiocarcinoma	36	36	16	20
COAD	Colon adenocarcinoma	478	453	239	214
DLBC	Lymphoid neoplasm diffuse large B-cell lymphoma	48	48	22	26
ESCA	Esophageal carcinoma	162	161	138	23
GBM	Glioblastoma multiforme	168	160	104	56
HNSC	Head and neck squamous cell carcinoma	502	499	366	133
KICH	Kidney chromophobe	65	64	38	26
KIRC	Kidney renal clear cell carcinoma	539	528	343	185
KIRP	Kidney renal papillary cell carcinoma	289	286	210	76
LAML	Acute myeloid leukemia	151	NA	NA	NA
LGG	Brain lower grade glioma	528	511	283	228
LIHC	Liver hepatocellular carcinoma	374	370	249	121
LUAD	Lung adenocarcinoma	535	504	234	270
LUSC	Lung squamous cell carcinoma	502	496	367	129
MESO	Mesothelioma	86	83	68	15
OV	Ovarian serous cystadenocarcinoma	379	374	NA	374
PAAD	Pancreatic adenocarcinoma	178	177	97	80
PCPG	Pheochromocytoma and paraganglioma	183	179	78	101
PRAD	Prostate adenocarcinoma	499	495	495	NA
READ	Rectum adenocarcinoma	166	165	90	75
SARC	Sarcoma	263	259	118	141
SKCM	Skin cutaneous melanoma	471	454	282	172
STAD	Stomach adenocarcinoma	375	372	239	133
TGCT	Testicular germ cell tumors	139	134	134	NA
THCA	Thyroid carcinoma	510	502	135	367
THYM	Thymoma	119	118	62	56
USEC	Uterine corpus endometrial carcinoma	552	542	NA	542
UCS	Uterine carcinosarcoma	56	56	NA	56
UVM	Uveal melanoma	80	79	44	35

*TCGA: The Cancer Genome Atlas; PFI: progression-free interval, a survival endpoint. Symbols highlighted in blue (male) and pink (female) denote sex-specific tumor types. No PFI data is available for LAML.
