# Supplementary material for: Elevated Tumor-Associated Androgen Receptor Activity Correlates with Poor Immune Infiltration and Immunotherapy Response across Cancer Types
Source: Cancer Res Commun. 2026 Jan 5;6(1):17–35. doi: 10.1158/2767-9764.CRC-25-0409 (PMC12766373; doi:10.1158/2767-9764.CRC-25-0409)
Supplement: Supplementary Table S2 — Abbreviations and evaluated tissue sample numbers in the GTEx database. [file crc-25-0409_supplementary_table_s2_suppst2.pdf]

Supplementary Table S2. Abbreviations and evaluated tissue sample numbers in the GTEx database

Abbreviation	Tissue Site Detail	Sample number (n)
ADPSBQ	Adipose - Subcutaneous	663
ADPVSC	Adipose - Visceral (Omentum)	541
ADRNLG	Adrenal Gland	258
ARTAORT	Artery - Aorta	432
ARTCRN	Artery - Coronary	240
ARTTBL	Artery - Tibial	663
BLDDER	Bladder	21
BRNAMY	Brain - Amygdala	152
BRNACC	Brain - Anterior cingulate cortex (BA24)	176
BRNCDT	Brain - Caudate (basal ganglia)	246
BRNCHB	Brain - Cerebellar Hemisphere [Frozen]	215
BRNCHA	Brain - Cerebellum [PAXgene]	241
BRNCTXA	Brain - Cortex [PAXgene]	255
BRNCTXB	Brain - Frontal Cortex (BA9) [Frozen]	209
BRNHPP	Brain - Hippocampus	197
BRNHPT	Brain - Hypothalamus	202
BRNNCC	Brain - Nucleus accumbens (basal ganglia)	246
BRNPTM	Brain - Putamen (basal ganglia)	205
BRNSPC	Brain - Spinal cord (cervical c-1)	159
BRNSNG	Brain - Substantia nigra	139
BREAST	Breast - Mammary Tissue	459
FIBRBLS	Cells - Cultured fibroblasts	504
LCL	Cells - EBV-transformed lymphocytes	174
CVXECT	Cervix - Ectocervix	9
CVSEND	Cervix - Endocervix	10
CLNSGM	Colon - Sigmoid	373
CLNTRN	Colon - Transverse	406
ESPGEJ	Esophagus - Gastroesophageal Junction	375
ESPMCS	Esophagus - Mucosa	555
ESPMSL	Esophagus - Muscularis	515
FLLPNT	Fallopian Tube	9
HRTAA	Heart - Atrial Appendage	429
HRTLV	Heart - Left Ventricle	432
KDNCTX	Kidney - Cortex	85
KDNMDL	Kidney - Medulla	4

Supplementary Table S2 (continued). Abbreviations and evaluated tissue sample numbers in the GTEx database

Abbreviation	Tissue Site Detail	Sample number (n)
LIVER	Liver	226
LUNG	Lung	578
SLVRYG	Minor Salivary Gland	162
MSCLSK	Muscle - Skeletal	803
NERVET	Nerve - Tibial	619
OVARY	Ovary	180
PNCREAS	Pancreas	328
PTTARY	Pituitary	283
PRSTTE	Prostate	245
SKINNS	Skin - Not Sun Exposed (Suprapubic)	604
SKINS	Skin - Sun Exposed (Lower leg)	701
SNTTRM	Small Intestine - Terminal Ileum	187
SPLEEN	Spleen	241
STMACH	Stomach	359
TESTIS	Testis	361
THYROID	Thyroid	653
UTERUS	Uterus	142
VAGINA	Vagina	156
WHLBLD	Whole Blood	755
