# Supplementary material for: Elevated Tumor-Associated Androgen Receptor Activity Correlates with Poor Immune Infiltration and Immunotherapy Response across Cancer Types
Source: Cancer Res Commun. 2026 Jan 5;6(1):17–35. doi: 10.1158/2767-9764.CRC-25-0409 (PMC12766373; doi:10.1158/2767-9764.CRC-25-0409)
Supplement: Supplementary Figure S1 — AR activity on overall survival outcome in pooled male and female samples across 33 TCGA cohorts. [file crc-25-0409_supplementary_figure_s1_suppsf1.pdf]

Supplementary Figure S1

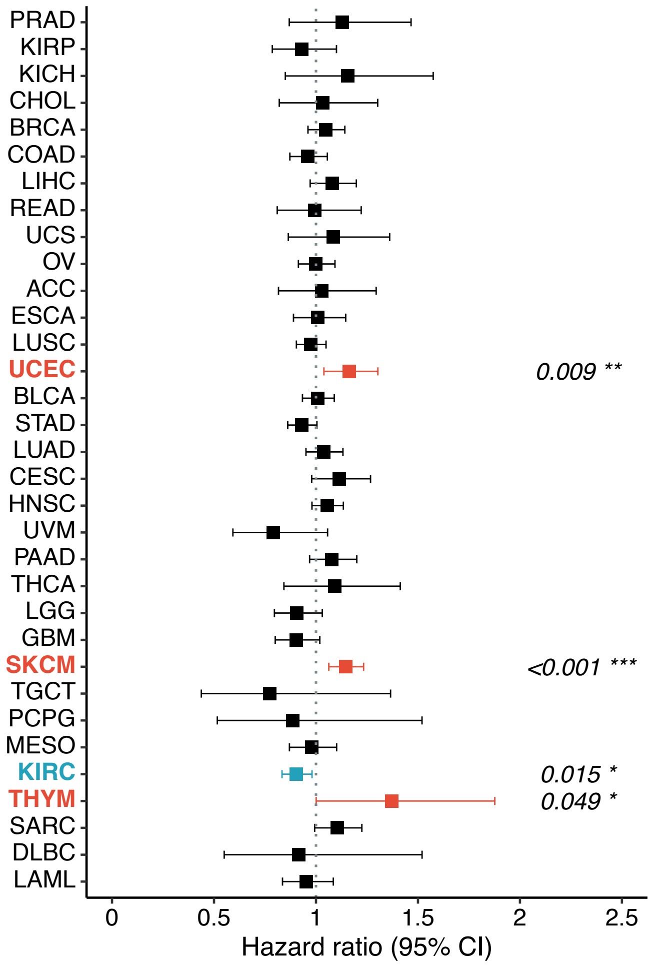

Supplementary Figure S1. AR activity on overall survival outcome in pooled male and female samples across 33 TCGA cohorts. Univariate Cox regression analysis of AR activity on overall survival endpoints was conducted across TCGA cohorts. Cancer types were ranked by decreasing median AR activity among the 33 TCGA cancer types (**Figure 1A**). Forest plots display hazard ratio (HR) estimates, 95% confidence intervals (CI), and corresponding *p*-values. Cancers where AR activity significantly correlates with a favorable prognosis are highlighted in dark cyan, while those significantly associated with a poorer prognosis are highlighted in red-orange. The data associated with the plot are provided in the Supplementary Data 3. Statistical significance: *, $P < 0.05$; **, $P < 0.01$; ***, $P < 0.001$.
