# Supplementary material for: Elevated Tumor-Associated Androgen Receptor Activity Correlates with Poor Immune Infiltration and Immunotherapy Response across Cancer Types
Source: Cancer Res Commun. 2026 Jan 5;6(1):17–35. doi: 10.1158/2767-9764.CRC-25-0409 (PMC12766373; doi:10.1158/2767-9764.CRC-25-0409)
Supplement: Supplementary Figure S3 — Association between AR/ESR1 ratio and progression-free interval (PFI) across TCGA cohorts. [file crc-25-0409_supplementary_figure_s3_suppsf3.pdf]

Supplementary Figure S3

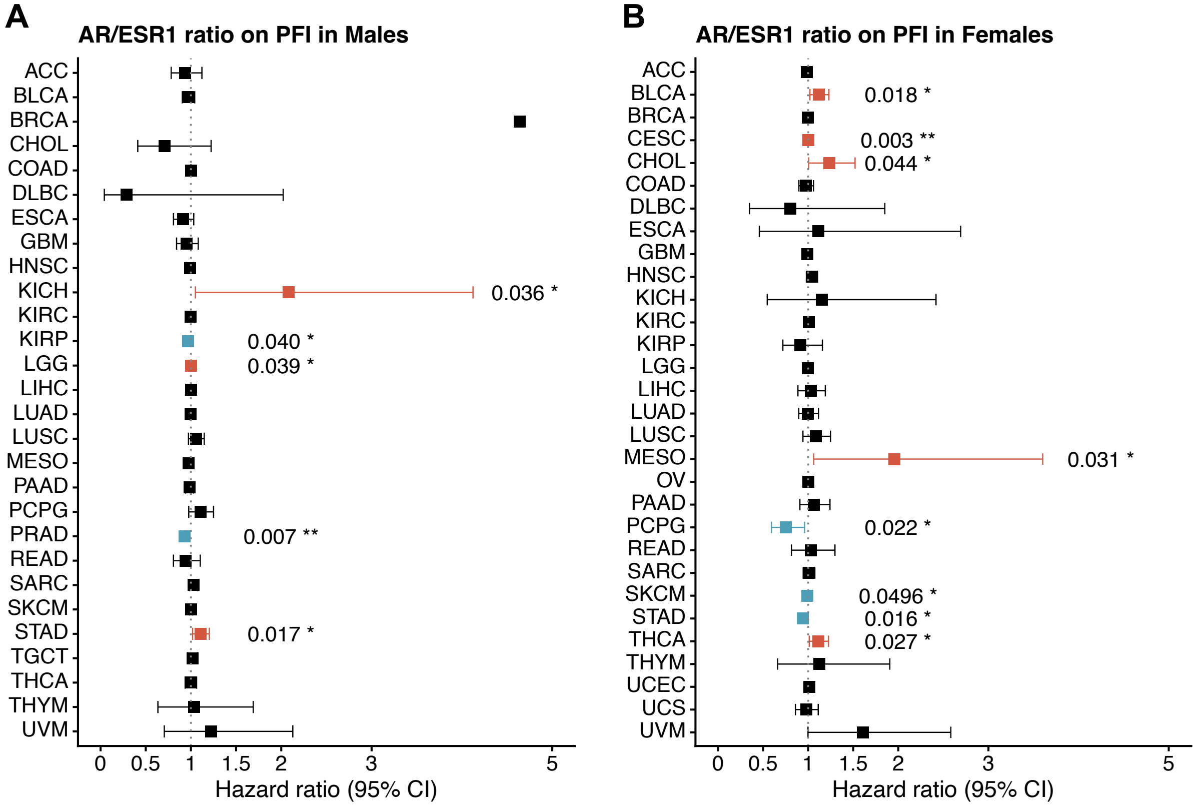

Supplementary Figure S3. Association between AR/ESR1 ratio and progression-free interval (PFI) across TCGA cohorts. A and B, Univariate Cox regression analysis of the AR/ERα activity ratio on PFI was performed across TCGA cancer types in males (A) and females (B). PFI data were not available for LAML. In panel (A), BRCA is shown without 95% CI bars due to the small sample size and resulting unreliable estimates. For some other cancer types, only a square box is visible because the lower and upper bounds of the 95% CI are extremely close to 1. Forest plots display hazard ratio (HR) estimates, 95% confidence intervals (CI), and corresponding *p*-values. Cancers in which ERα activity significantly correlates with a favorable prognosis are highlighted in dark cyan, while those associated with a poorer prognosis are highlighted in red-orange. The complete data underlying these plots are provided in Supplementary Data 4. PFI, progression-free interval; ESR1, estrogen receptor alpha (ERα). Statistical significance: *, $P < 0.05$; **, $P < 0.01$; ***, $P < 0.001$.
