# Supplementary material for: Elevated Tumor-Associated Androgen Receptor Activity Correlates with Poor Immune Infiltration and Immunotherapy Response across Cancer Types
Source: Cancer Res Commun. 2026 Jan 5;6(1):17–35. doi: 10.1158/2767-9764.CRC-25-0409 (PMC12766373; doi:10.1158/2767-9764.CRC-25-0409)
Supplement: Supplementary Figure S4 — Overview of ERβ activity and association with progression-free interval outcomes across TCGA cohorts. [file crc-25-0409_supplementary_figure_s4_suppsf4.pdf]

# Supplementary Figure S4

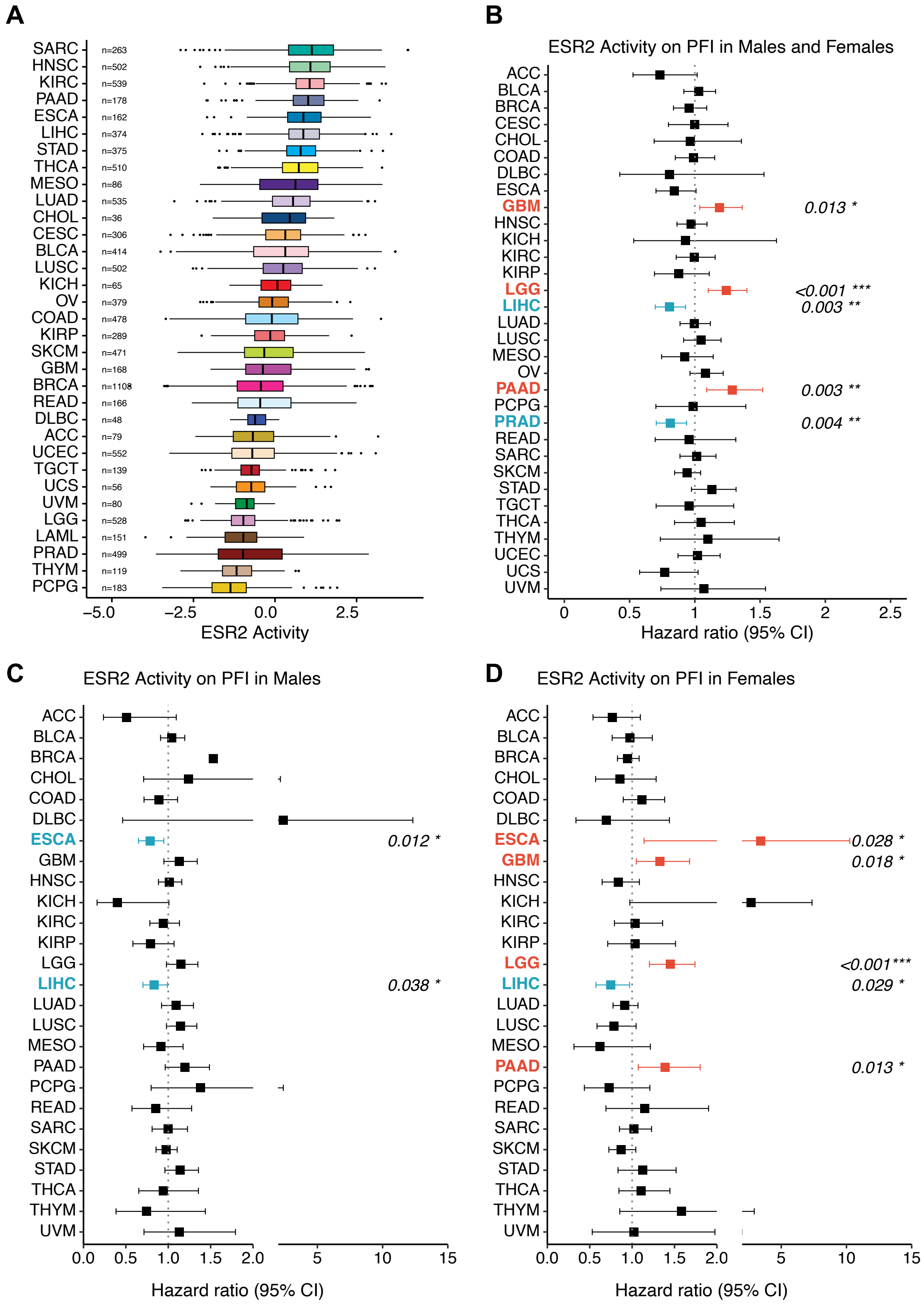

**Supplementary Figure S4. Overview of ERβ activity and association with progression-free interval outcomes across TCGA cohorts.** A, Boxplot displaying ERβ activity ranked in order of decreasing median ERβ activity among 33 TCGA cancer types. Values on the left correspond to the total number of tumor samples (n) analyzed in each cancer cohort. TCGA study abbreviations and sample sizes for each dataset are listed in Supplementary Table S1. The center line indicates the median, the bounds of the box indicate the upper and lower quartiles, the whiskers indicate the minimum and maximum, and outliers are marked with dots. B-D, Univariate Cox regression analysis of ERβ activity on PFI endpoints was conducted across TCGA , including pooled males and females (B), males only (C), and females only (D). There is no PFI data provided for LAML. Figure S4C and S4D include cancer types with both male and female tumor samples. Note that in Figure S4C, BRCA is not shown with the 95% CI due to the unreliability of the results caused by the small sample size. Forest plots display hazard ratio (HR) estimates, 95% confidence intervals (CI), and corresponding *p*-values. Cancers where ERβ activity significantly correlates with a favorable prognosis are highlighted in dark cyan, while those significantly associated with a poorer prognosis are highlighted in red-orange. The data associated with the plots are provided in the Supplementary Data 5. PFI: progression-free interval. ESR2: estrogen receptor beta (ERβ). Statistical significance: *, $P < 0.05$; **, $P < 0.01$; ***, $P < 0.001$.
