# Supplementary material for: Elevated Tumor-Associated Androgen Receptor Activity Correlates with Poor Immune Infiltration and Immunotherapy Response across Cancer Types
Source: Cancer Res Commun. 2026 Jan 5;6(1):17–35. doi: 10.1158/2767-9764.CRC-25-0409 (PMC12766373; doi:10.1158/2767-9764.CRC-25-0409)
Supplement: Supplementary Figure S6 — Correlation between AR activity and immune cell enrichment across 33 cancer types. [file crc-25-0409_supplementary_figure_s6_suppsf6.pdf]

Supplementary Figure S6

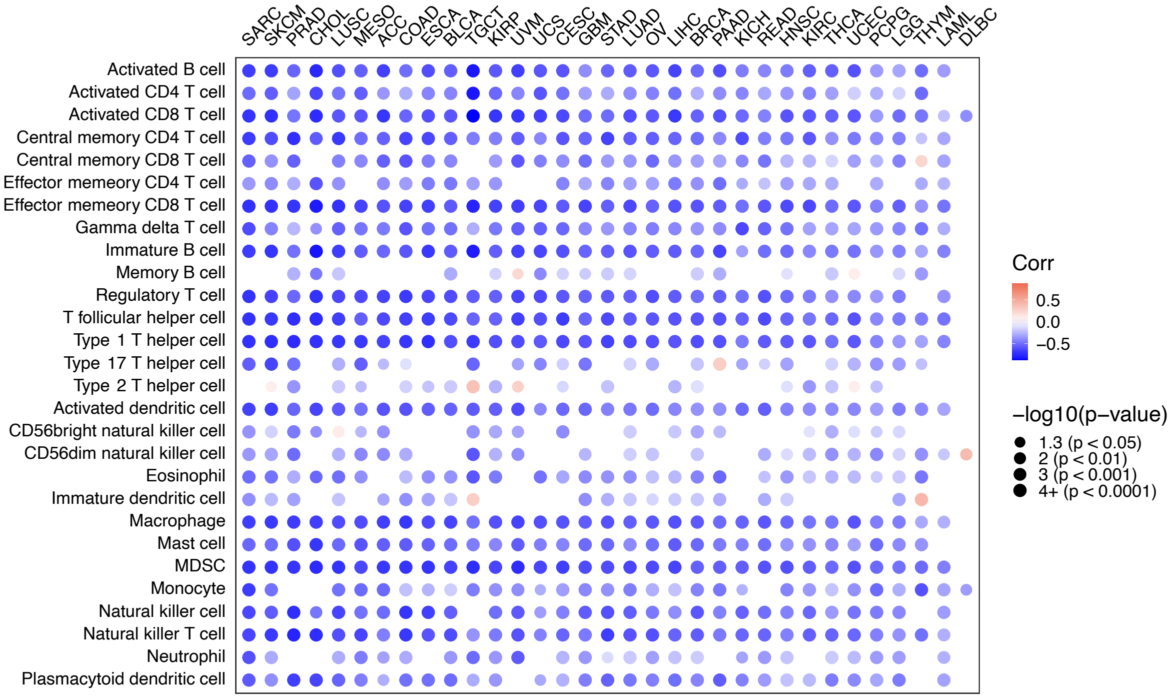

Supplementary Figure S6. Correlation between AR activity and immune cell enrichment across 33 cancer types. Correlation between AR activity and ssGSEA enrichment scores of 28 immune cell types across 33 cancer types. The *p*-value shown was determined using a two-tailed Pearson correlation. Positive correlation coefficients are displayed in orange, and negative correlation coefficients in blue. The color intensity is proportional to the correlation coefficients. The circle size corresponds to the *p*-values, while correlation coefficients with *p*-values > 0.05 are left blank. ssGSEA: single-sample Gene Set Enrichment Analysis.
