# Supplementary material for: Elevated Tumor-Associated Androgen Receptor Activity Correlates with Poor Immune Infiltration and Immunotherapy Response across Cancer Types
Source: Cancer Res Commun. 2026 Jan 5;6(1):17–35. doi: 10.1158/2767-9764.CRC-25-0409 (PMC12766373; doi:10.1158/2767-9764.CRC-25-0409)
Supplement: Supplementary Figure S7 — Correlations between ERa, ERb, and PR activity with six immune cell populations in all tumor samples across 32 TCGA cancer types. [file crc-25-0409_supplementary_figure_s7_suppsf7.pdf]

A

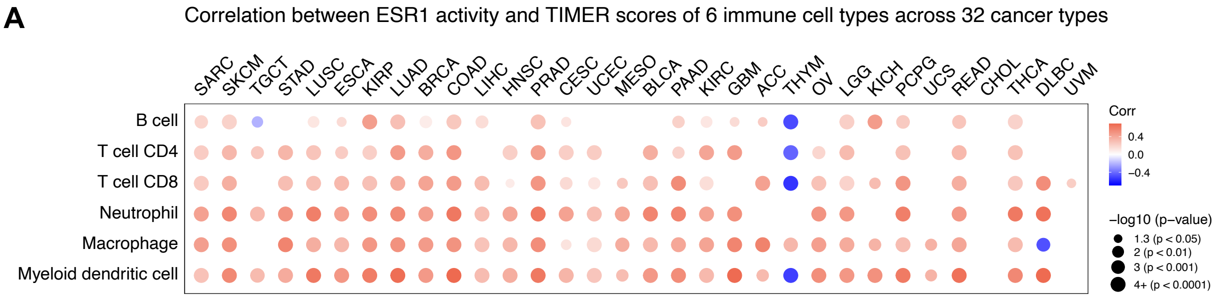

B

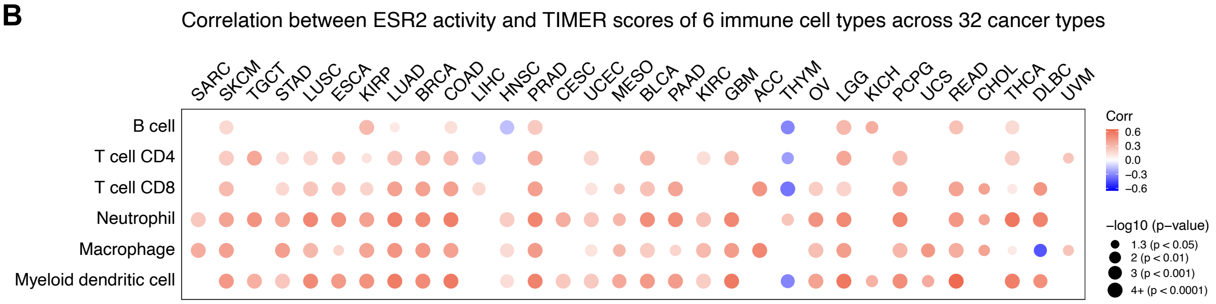

C

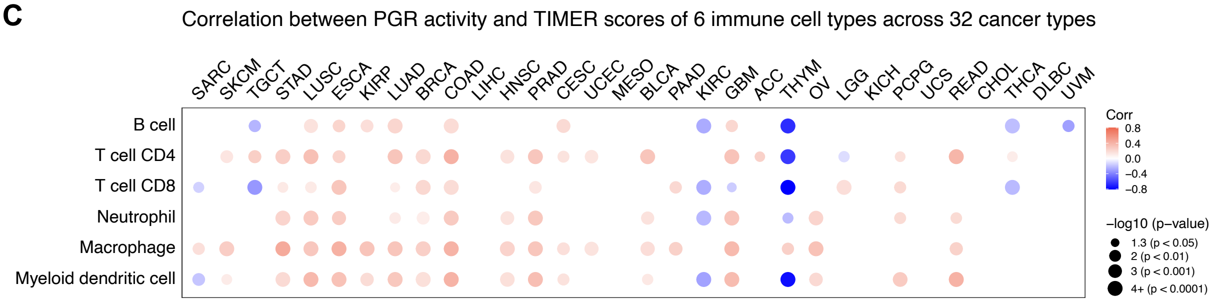

Supplementary Figure S7. Correlations between ERα, ERβ, and PR activity with six immune cell populations in all tumor samples across 32 TCGA cancer types. Dot plots illustrate the correlations between (A) estrogen receptor 1 (ERα; ESR1), (B) estrogen receptor 2 (ERβ; ESR2), and (C) progesterone receptor (PGR) and six immune cell populations (B cells, $CD4^+$ T cells, $CD8^+$ T cells, macrophages, dendritic cells, and neutrophils) across 32 cancer types in TCGA cohorts (LAML is not applicable in TIMER calculation). Each circle represents a correlation coefficient value analyzed using a two-tailed Pearson correlation test. Positive correlation coefficients are displayed in orange, and negative correlation coefficients are displayed in blue. The color intensity is proportional to the correlation coefficients. The circle size is proportional to the *p*-values, while correlation coefficients with *p*-values > 0.05 are left blank. The cancer types in the column are arranged in the same order as illustrated in Figure 3A. The enrichment scores of immune infiltration levels are determined using TIMER algorithm.
