# Supplementary material for: Elevated Tumor-Associated Androgen Receptor Activity Correlates with Poor Immune Infiltration and Immunotherapy Response across Cancer Types
Source: Cancer Res Commun. 2026 Jan 5;6(1):17–35. doi: 10.1158/2767-9764.CRC-25-0409 (PMC12766373; doi:10.1158/2767-9764.CRC-25-0409)
Supplement: Supplementary Figure S8 — Comparing nuclear receptor activity between males and females in 27 cancer types. [file crc-25-0409_supplementary_figure_s8_suppsf8.pdf]

Supplementary Figure S8

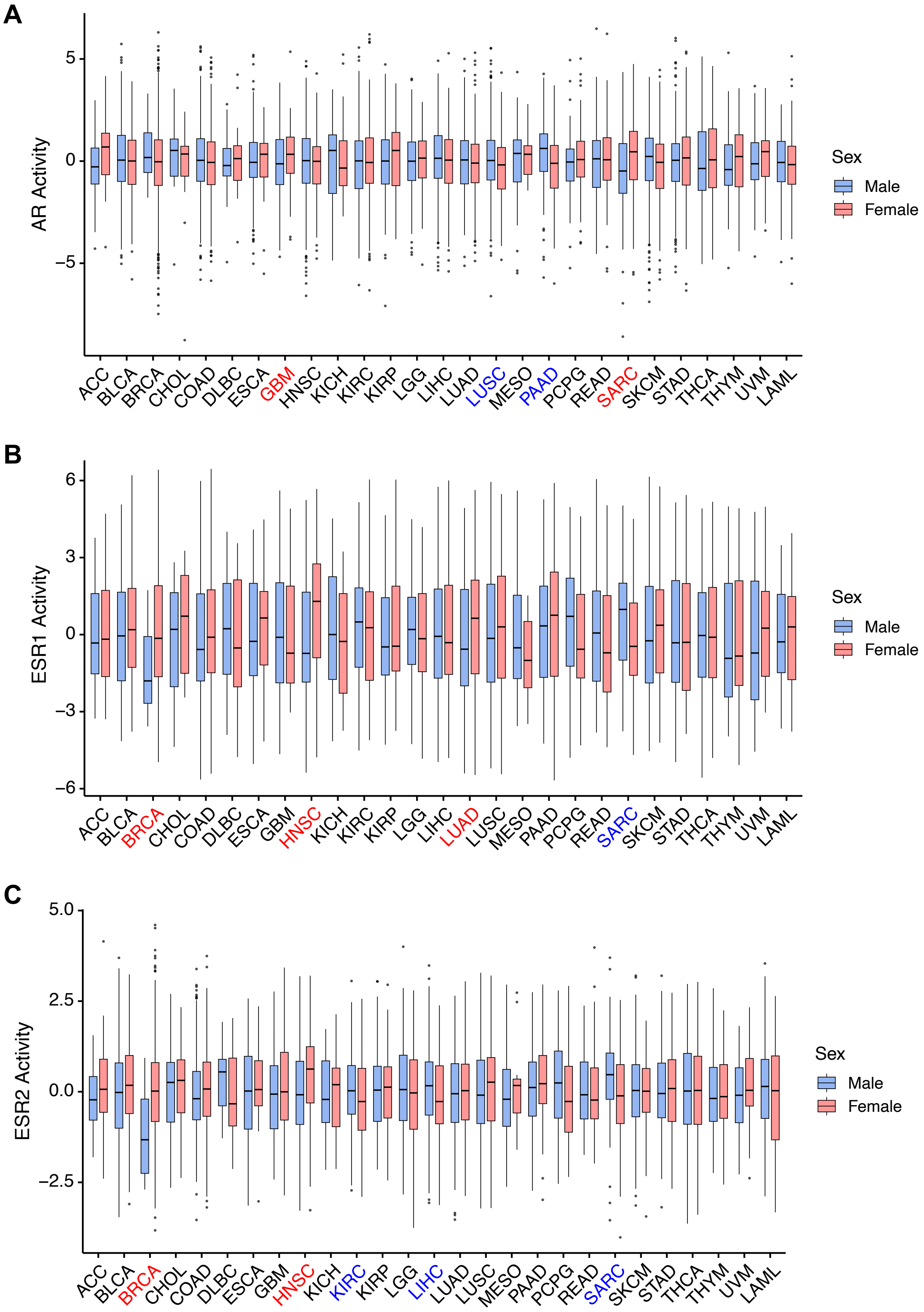

Supplementary Figure S8 (continued)

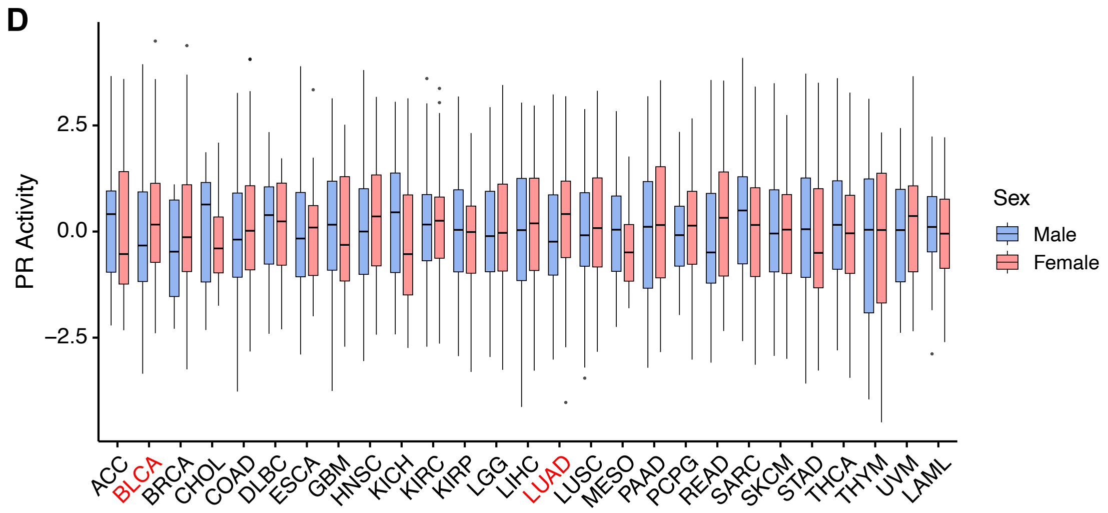

Supplementary Figure S8. Comparing nuclear receptor activity between males and females in 27 cancer types. A-D, Boxplots illustrating AR (A), ERα (B), ERβ (C), and PR (D) activity in 27 cancer types with both male and female tumor samples. Blue text represents activity that is significantly higher in males than in females, while pink text represents activity that is significantly higher in females than in males. Statistical significance was calculated using two-sided Wilcoxon rank-sum tests between males and females. The center line indicates the median, the bounds of the box indicate the upper and lower quartiles, the whiskers indicate the minimum and maximum values, and outliers are marked with dots. ESR1: estrogen receptor alpha (ERα). ESR2: estrogen receptor beta (ERβ).
