# Supplementary material for: Elevated Tumor-Associated Androgen Receptor Activity Correlates with Poor Immune Infiltration and Immunotherapy Response across Cancer Types
Source: Cancer Res Commun. 2026 Jan 5;6(1):17–35. doi: 10.1158/2767-9764.CRC-25-0409 (PMC12766373; doi:10.1158/2767-9764.CRC-25-0409)
Supplement: Supplementary Figure S9 — Correlations between AR activity and six immune cell populations in males and females. [file crc-25-0409_supplementary_figure_s9_suppsf9.pdf]

Supplementary Figure S9

A

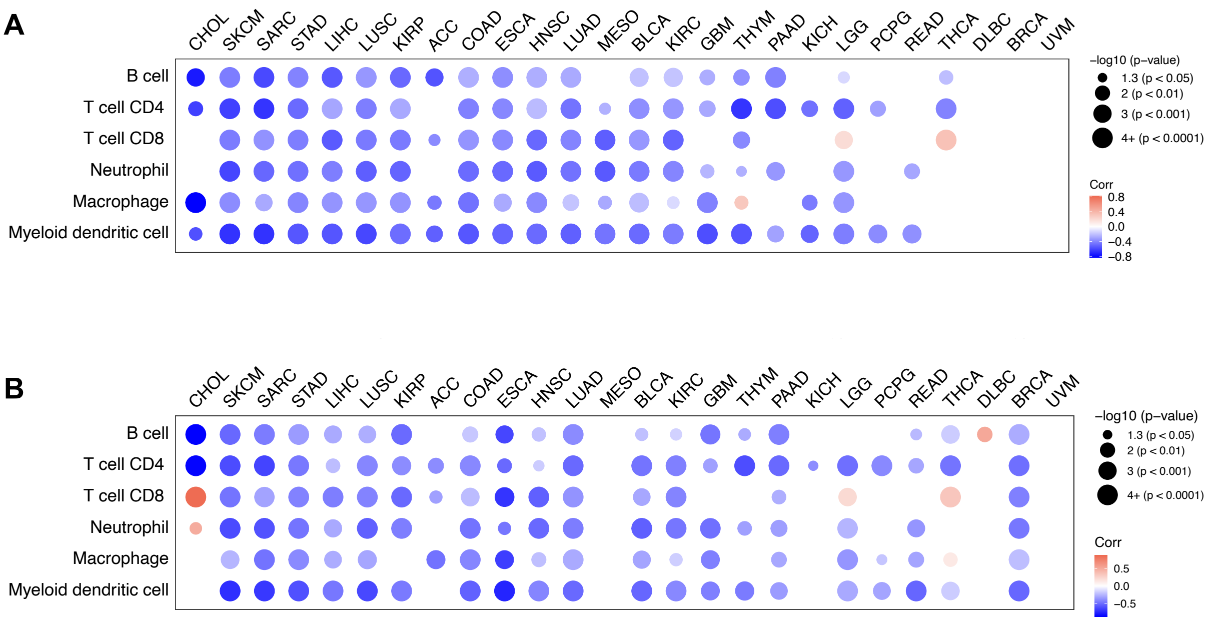

B

Supplementary Figure S9. Correlations between AR activity and six immune cell populations in males and females. Dots plots showing correlations between AR activity and B cells, CD4+ T cells, CD8+ T cells, macrophages, dendritic cells, and neutrophils in males (A) and females (B) across 26 TCGA cancer types. Each circle represents a correlation coefficient value analyzed by two-tailed Pearson correlation test. Positive correlation coefficients are displayed in orange color and negative correlation coefficients in blue color. The color intensity is proportional to the correlation coefficients. The circle size is proportional to the *p*-values, while correlation coefficients with *p*-values > 0.05 are blank. Cancer types (column) are sorted in order of decreasing mean of correlation coefficient. The enrichment scores of immune infiltration levels are determined by TIMER algorithm.
