# Supplementary material for: Elevated Tumor-Associated Androgen Receptor Activity Correlates with Poor Immune Infiltration and Immunotherapy Response across Cancer Types
Source: Cancer Res Commun. 2026 Jan 5;6(1):17–35. doi: 10.1158/2767-9764.CRC-25-0409 (PMC12766373; doi:10.1158/2767-9764.CRC-25-0409)
Supplement: Supplementary Figure S10 — Venn Diagram of Immune Signatures. [file crc-25-0409_supplementary_figure_s10_suppsf10.pdf]

Supplementary Figure S10

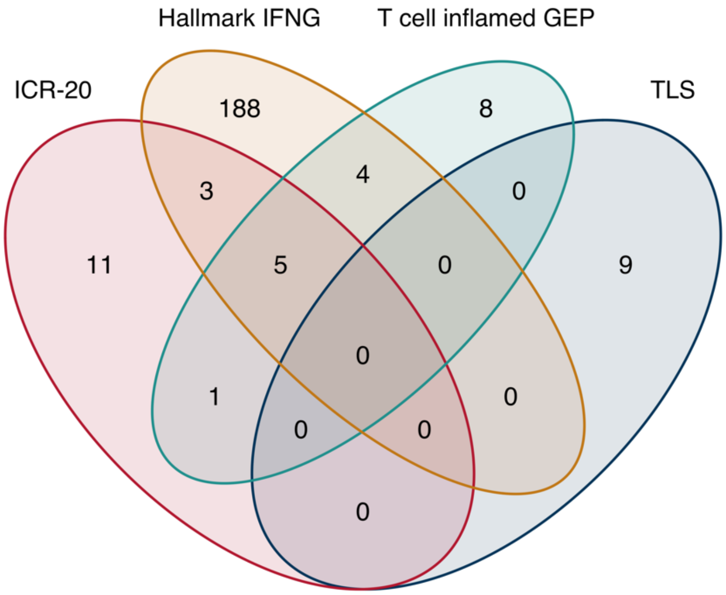

Supplementary Figure S10. Venn Diagram of Immune Signatures. This Venn diagram illustrates the overlap between four immune-related gene signatures: Hallmark interferon-gamma signaling (IFN-γ), the T cell-inflamed gene expression profile (GEP), the immunologic constant of rejection (ICR)-20 gene signature, and the TLS signature. Full details of the gene lists are provided in Supplementary Data 1.
