# Supplementary material for: Elevated Tumor-Associated Androgen Receptor Activity Correlates with Poor Immune Infiltration and Immunotherapy Response across Cancer Types
Source: Cancer Res Commun. 2026 Jan 5;6(1):17–35. doi: 10.1158/2767-9764.CRC-25-0409 (PMC12766373; doi:10.1158/2767-9764.CRC-25-0409)
Supplement: Supplementary Figure S11 — Correlations between AR activity and immune signatures with overlapping genes removed. [file crc-25-0409_supplementary_figure_s11_suppsf11.pdf]

**Supplementary Figure S11**

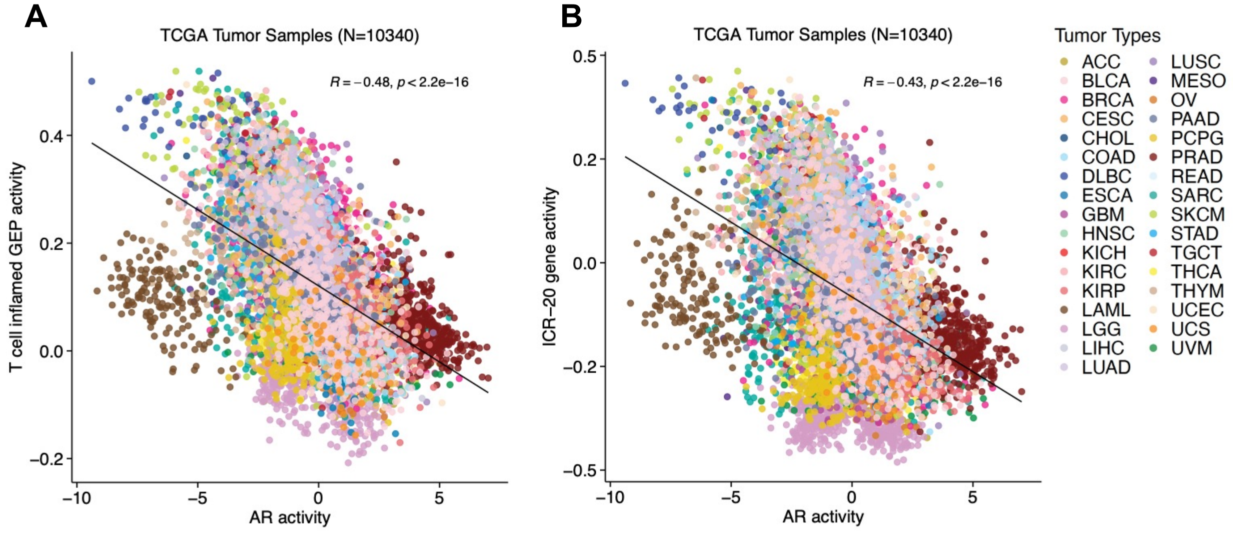


**Supplementary Figure S11.** Correlations between AR activity and immune signatures with overlapping genes removed. Scatter plots (A, B) showing the Pearson correlation of AR activity with (A) T cell–inflamed GEP and (B) ICR-20 gene signature activity scores of all TCGA tumor samples. The ssGSEA scores of immune signatures are calculated by removing 5 overlapping genes between the T cell-inflamed GEP and ICR-20 signatures. Each dot represents one tumor sample (n = 10,340), with colors indicating different tumor types. Tumor types are listed on the right by color code (n = 33). T cell-inflamed gene expression profile (GEP), immunologic constant of rejection (ICR)-20 gene signature.
