# Supplementary material for: Elevated Tumor-Associated Androgen Receptor Activity Correlates with Poor Immune Infiltration and Immunotherapy Response across Cancer Types
Source: Cancer Res Commun. 2026 Jan 5;6(1):17–35. doi: 10.1158/2767-9764.CRC-25-0409 (PMC12766373; doi:10.1158/2767-9764.CRC-25-0409)
Supplement: Supplementary Figure S13 — ERb activity levels are positively correlated with gene signatures of three prognostic gene signatures and TLS across 33 TCGA cohorts. [file crc-25-0409_supplementary_figure_s13_suppsf13.pdf]

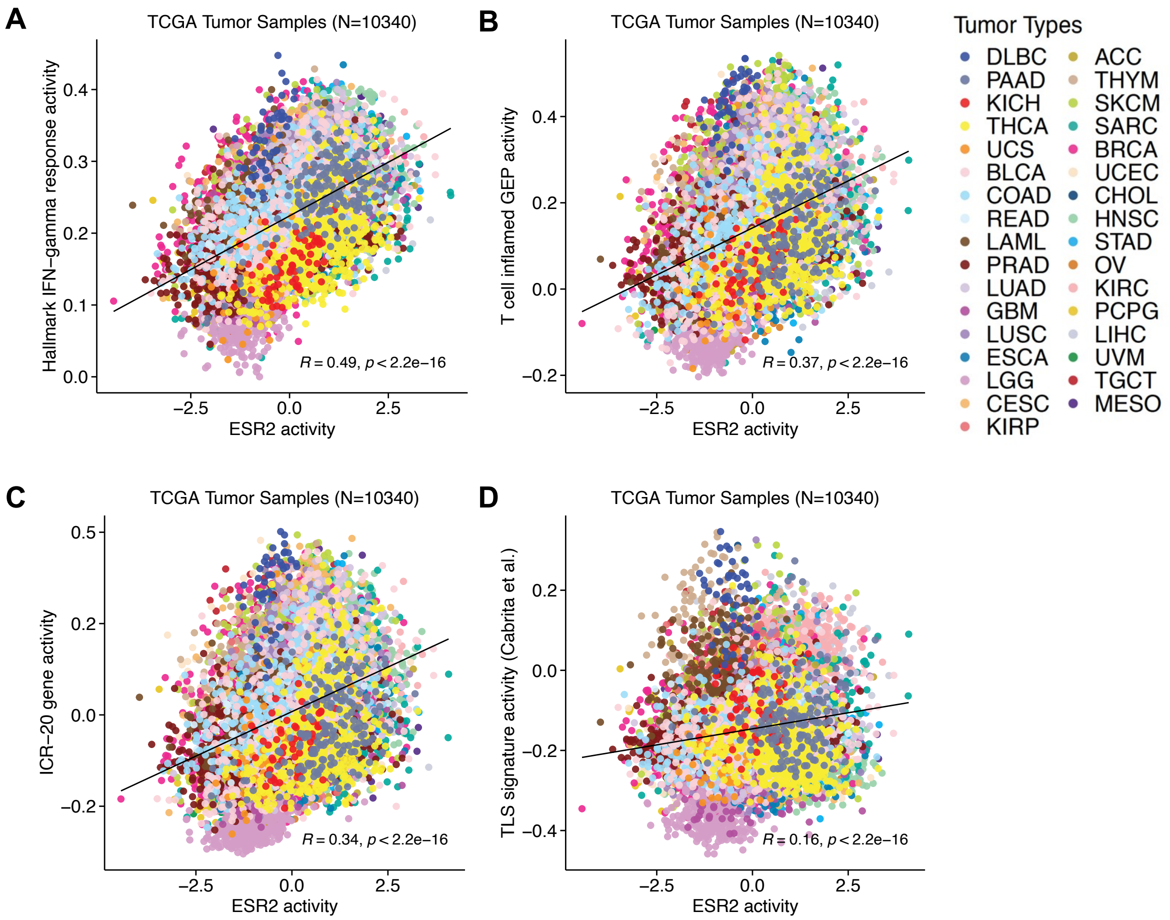

Supplementary Figure S13. ERβ activity levels are positively correlated with gene signatures of three prognostic gene signatures and TLS across 33 TCGA cohorts. Scatter plots (A-D) showing the Pearson correlation of ERα activity with A, Hallmark IFN-γ pathway; B, T cell–inflamed GEP; C, ICR-20 gene; and D, TLS signature activity scores of all TCGA tumor samples. Each dot represents one tumor sample (n=10,340), with colors indicating different tumor types. Tumor types are listed on the right by color code (n = 33). GEP: gene expression profile. ICR: immunologic constant of rejection. TLS: tertiary lymphoid structures. ns: non-significant. ESR2: estrogen receptor beta (ERβ). Tumor types in the figure legend, listed from left to right, are ordered by descending correlation coefficient, with DLBC showing the most positive correlation between ESR2 activity and Hallmark IFN-γ response activity.
