# Supplementary material for: Elevated Tumor-Associated Androgen Receptor Activity Correlates with Poor Immune Infiltration and Immunotherapy Response across Cancer Types
Source: Cancer Res Commun. 2026 Jan 5;6(1):17–35. doi: 10.1158/2767-9764.CRC-25-0409 (PMC12766373; doi:10.1158/2767-9764.CRC-25-0409)
Supplement: Supplementary Figure S14 — Correlation between PR activity and with gene signatures of three prognostic gene signatures and TLS across 33 TCGA cohorts. [file crc-25-0409_supplementary_figure_s14_suppsf14.pdf]

Supplementary Figure S14

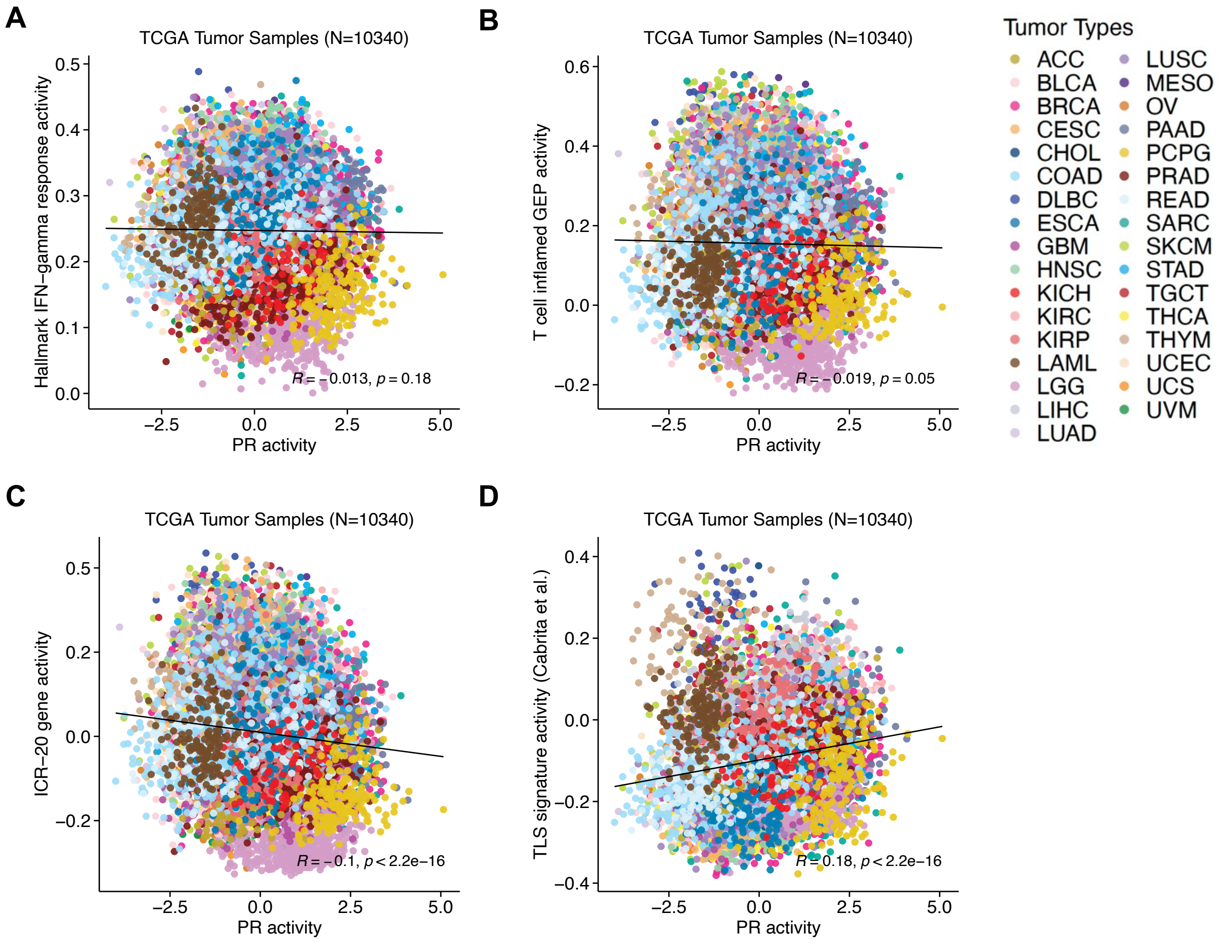

Supplementary Figure S14. Correlation between PR activity and with gene signatures of three prognostic gene signatures and TLS across 33 TCGA cohorts. Scatter plots (A-D) showing the Pearson correlation of ERα activity with A, Hallmark IFN-γ pathway; B, T cell–inflamed GEP; C, ICR-20 gene; and D, TLS signature activity scores of all TCGA tumor samples. Each dot represents one tumor sample (n=10,340), with colors indicating different tumor types. Tumor types are listed on the right by color code (n = 33). GEP: gene expression profile. ICR: immunologic constant of rejection. TLS: tertiary lymphoid structures. ns: non-significant. PR: progesterone receptor. Tumor types in the figure legend, listed from left to right, are ordered alphabetically.
