# Supplementary material for: Elevated Tumor-Associated Androgen Receptor Activity Correlates with Poor Immune Infiltration and Immunotherapy Response across Cancer Types
Source: Cancer Res Commun. 2026 Jan 5;6(1):17–35. doi: 10.1158/2767-9764.CRC-25-0409 (PMC12766373; doi:10.1158/2767-9764.CRC-25-0409)
Supplement: Supplementary Figure S15 — AR activity levels are negatively correlated with gene signatures of IFN-γ signaling and TLS in GTEx tissue samples. [file crc-25-0409_supplementary_figure_s15_suppsf15.pdf]

Supplementary Figure S15

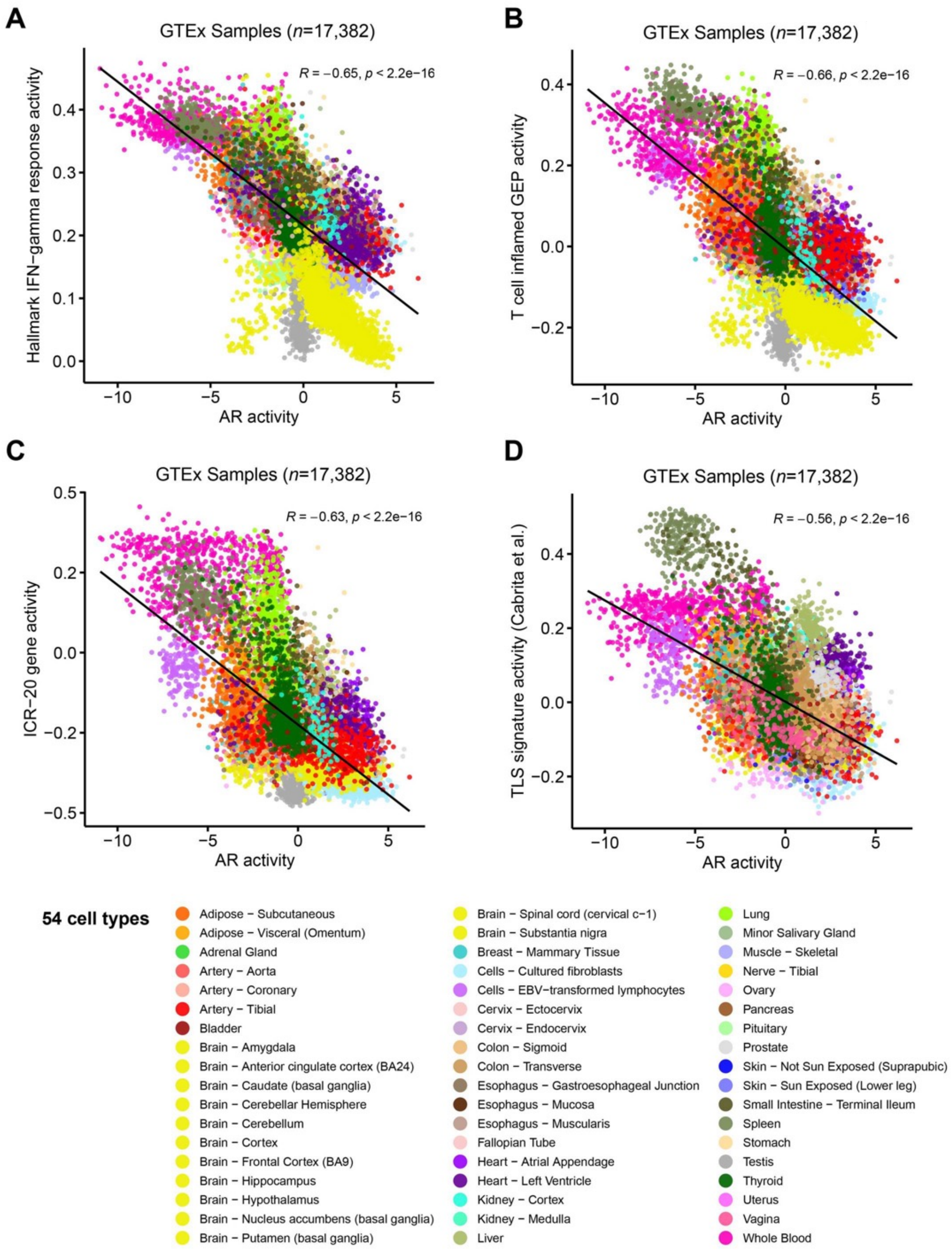

Supplementary Figure S15. AR activity levels are negatively correlated with gene signatures of IFN-γ signaling and TLS in GTEx tissue samples. Scatter plots (A-D) showing the Pearson correlation of AR activity with A, Hallmark IFN-γ pathway; B, T cell–inflamed GEP, C, ICR 20-gene; and D, TLS signature activity scores of all GTEx tissue samples. Each dot represents one normal tissue sample (*n*=17,382), with colors indicating 54 different tissue types. Tissue types are listed in the legend by color code. GEP: gene expression profile. ICR: immunologic constant of rejection. TLS: tertiary lymphoid structures. GTEx: The Genotype-Tissue Expression.
