# Supplementary material for: Elevated Tumor-Associated Androgen Receptor Activity Correlates with Poor Immune Infiltration and Immunotherapy Response across Cancer Types
Source: Cancer Res Commun. 2026 Jan 5;6(1):17–35. doi: 10.1158/2767-9764.CRC-25-0409 (PMC12766373; doi:10.1158/2767-9764.CRC-25-0409)
Supplement: Supplementary Figure S16 — Overview of AR Activity across 54 GTEx tissue types. Boxplots displaying AR activity ranked in order of decreasing AR activity median among 54 GTEx tissue types. [file crc-25-0409_supplementary_figure_s16_suppsf16.pdf]

Supplementary Figure S16

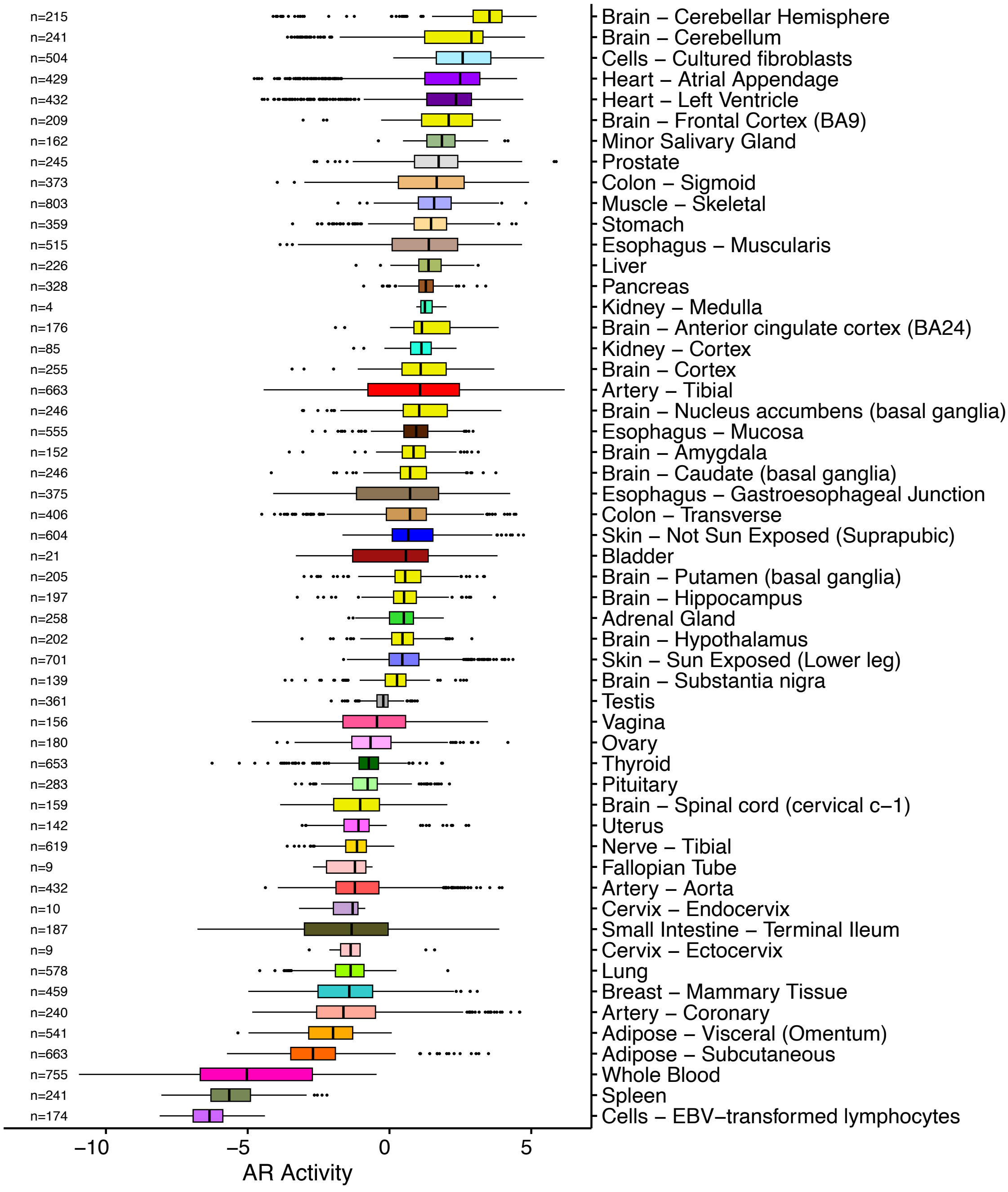

Supplementary Figure S16. Overview of AR Activity across 54 GTEx tissue types. Boxplots displaying AR activity ranked in order of decreasing AR activity median among 54 GTEx tissue types. Values on the left correspond to the total number of tissue samples (n) analyzed in each tissue cohort. The center line indicates median, bounds of the box indicate upper and lower quartiles, whiskers indicate minimum and maximum, and outliers are marked with dots. GTEx: The Genotype-Tissue Expression.
