# Supplementary material for: Elevated Tumor-Associated Androgen Receptor Activity Correlates with Poor Immune Infiltration and Immunotherapy Response across Cancer Types
Source: Cancer Res Commun. 2026 Jan 5;6(1):17–35. doi: 10.1158/2767-9764.CRC-25-0409 (PMC12766373; doi:10.1158/2767-9764.CRC-25-0409)
Supplement: Supplementary Figure S17 — Correlations between activity and immune signature scores in tissue types from the GTEx database. [file crc-25-0409_supplementary_figure_s17_suppsf17.pdf]

Supplementary Figure S17

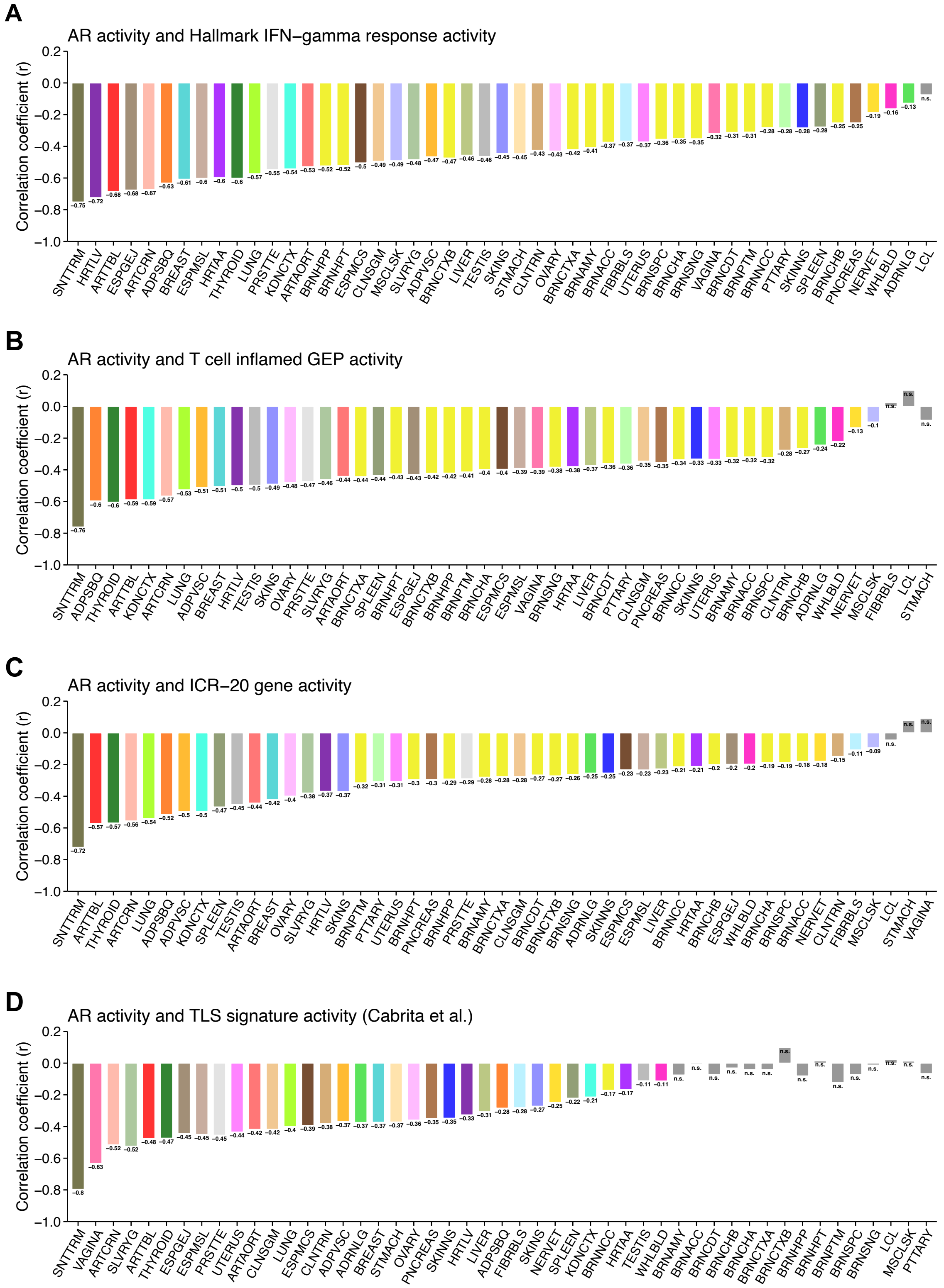

Supplementary Figure S17. Correlations between activity and immune signature scores in tissue types from the GTEx database. A-D, Bar plots displaying the Pearson correlation of AR activity with four parameters: A) Hallmark IFN-γ pathway, B) T cell–inflamed GEP, C) ICR 20-gene, and D) TLS signature activity scores within each tissue type from the GTEx database. Tissue types with fewer than 30 samples were excluded from the analysis. The tissue type abbreviations and sample numbers are listed in Supplementary Table S2. The X-axis represents tissue types arranged in descending order of correlation coefficient values (Y-axis). The text below each bar indicates the Pearson correlation coefficients. Bars on the far right with a *p*-value greater than 0.05 are colored in light grey. Definitions: GEP: Gene Expression Profile, ICR: Immunologic Constant of Rejection, TLS: Tertiary Lymphoid Structures, GTEx: Genotype-Tissue Expression. 'ns' indicates non-significant correlations.
