# Supplementary material for: Elevated Tumor-Associated Androgen Receptor Activity Correlates with Poor Immune Infiltration and Immunotherapy Response across Cancer Types
Source: Cancer Res Commun. 2026 Jan 5;6(1):17–35. doi: 10.1158/2767-9764.CRC-25-0409 (PMC12766373; doi:10.1158/2767-9764.CRC-25-0409)
Supplement: Supplementary Figure S18 — AR activity levels are negatively correlated with immune cell infiltration in TME of human PCa evaluated by scRNA-seq. [file crc-25-0409_supplementary_figure_s18_suppsf18.pdf]

Supplementary Figure S18

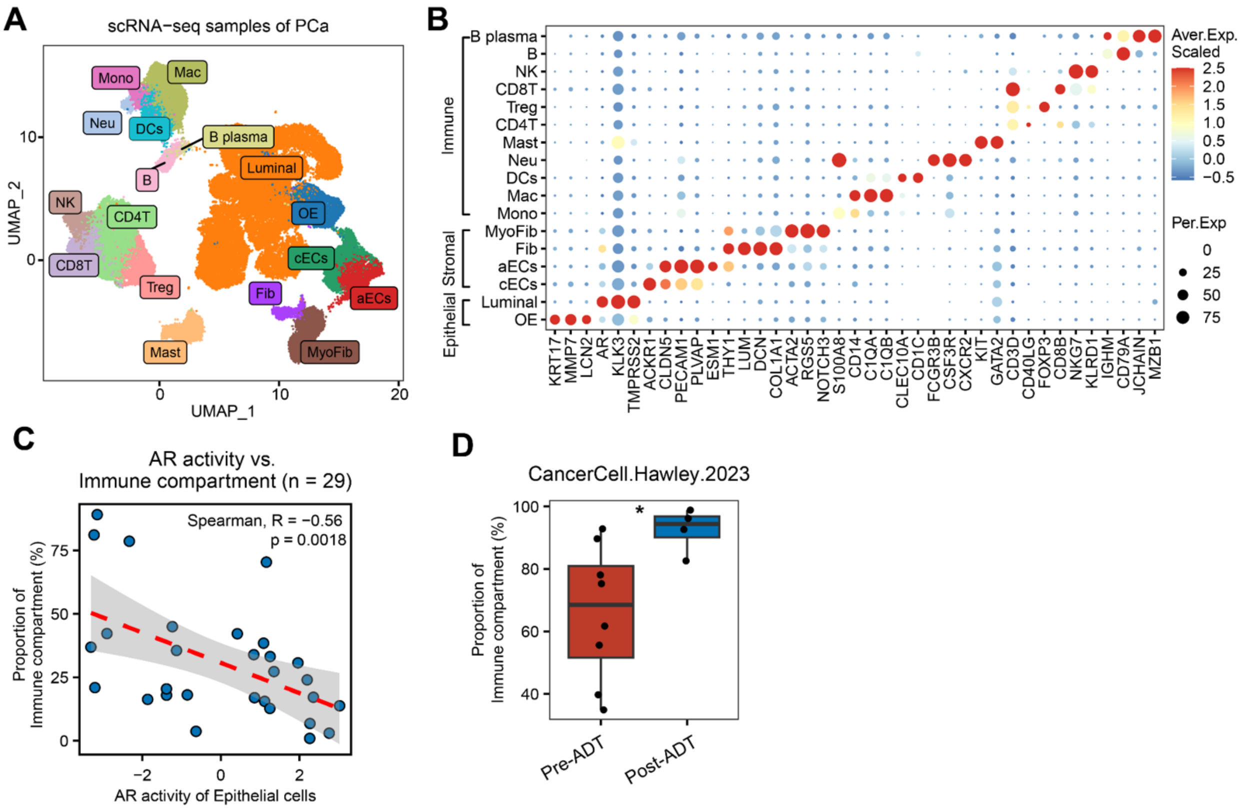

Supplementary Figure S18. AR activity levels are negatively correlated with immune cell infiltration in TME of human PCa evaluated by scRNA-seq. A, UMAP showing the distribution of 17 major cell types in a scRNA-seq atlas of human PCa (tumor samples = 29, cells = 79,830). B, Dot plot showing representative marker genes for each cell type. C, Spearman correlation analysis between AR activity of epithelial cells and the proportion of leukocytes within the total cell population (y-axis) in each sample from the scRNA-seq meta-atlas. D, Box plots showing the level of immune cell infiltration between pre-ADT and post-ADT PCa samples from the human PCa scRNA-seq dataset (PCa samples = 12, cells = 31,231) that was generated by Hawley et al. Each dot represents one PCa sample. *P < 0.05, Wilcoxon rank-sum test.
