# Supplementary material for: Elevated Tumor-Associated Androgen Receptor Activity Correlates with Poor Immune Infiltration and Immunotherapy Response across Cancer Types
Source: Cancer Res Commun. 2026 Jan 5;6(1):17–35. doi: 10.1158/2767-9764.CRC-25-0409 (PMC12766373; doi:10.1158/2767-9764.CRC-25-0409)
Supplement: Supplementary Figure S19 — Correlation analysis of ERα and PR expression with immune cell markers CD45 (A, B) and CD4 (C, D) in Digital Spatial Profiling protein data across breast, ovarian, and sarcoma cancer types. [file crc-25-0409_supplementary_figure_s19_suppsf19.pdf]

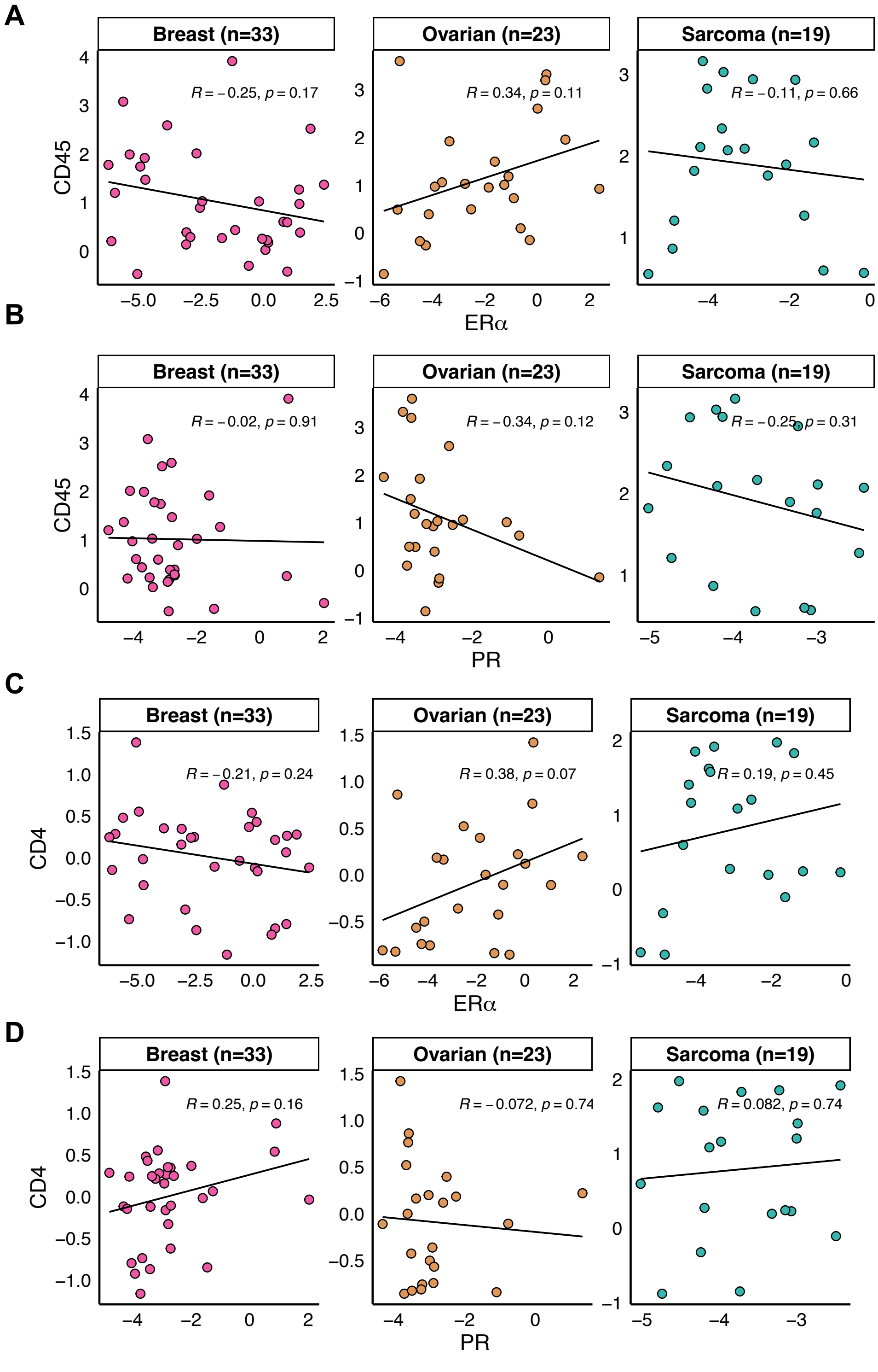

Supplementary Figure S19. Correlation analysis of ERα and PR expression with immune cell markers CD45 (A, B) and CD4 (C, D) in Digital Spatial Profiling protein data across breast, ovarian, and sarcoma cancer types.
